# Supplementary material for: Barriers to evidence-based acute stroke care in Ghana: a qualitative study on the perspectives of stroke care professionals
Source: BMJ Open. 2017 Apr 27;7(4):e015385. doi: 10.1136/bmjopen-2016-015385 (PMC5719663; doi:10.1136/bmjopen-2016-015385)
Supplement: Supplementary data [file bmjopen-2016-015385supp001.pdf]

Supporting File 1: Consolidated criteria for reporting qualitative studies (COREQ): 32-item checklist

No. Item	Guide questions/description	Reported on Page #
**Domain 1**: **Research team and reflexivity**		
Personal Characteristics		
1. Interviewer/facilitator	LB and AdGA facilitated the recruitment process. LB conducted the interviews	Page 7<br>Line 175-176, 186,
2. Credentials	LB (PhD Candidate), AdGA (PhD), GM (PhD), AS (PhD), CKYC (PhD), SS (PhD)	Page 6-7<br>Lines 155-164
3. Occupation	LB (Public Health PhD Candidate), AdGA (Professor of Social Psychology), AS (Health Services Researcher and Public Health Lecturer), GM (Epidemiologist and Public Health Lecturer), CKYC (Senior Lecturer in Health Psychology, Knowledge dissemination and implementation researcher) SS (Associate Professor of Public Health)	Page 6-7<br>Lines 155-165
4. Gender	Four researchers were males and two were females	Page 7<br>Lines 165-166
5. Experience and training	LB, AdGA, and AS have extensive experience in conducting qualitative research studies. GM, CKYC, SS have relevant training and knowledge in qualitative research studies.	Page 6-7<br>Lines 155-164
Relationship with participants		
6. Relationship established	No	Page 6-7<br>Lines 155-166
7. Participant knowledge of the interviewer	The interviewer (LB) is familiar with some of the study contexts through previous works so it is possible some participants have met or are aware of their works.	Page 6<br>Lines 155-158
8. Interviewer characteristics	The interviewer (LB) is a health services researcher with interest in health services and policy research, research on implementation science and quality improvement interventions for stroke care health professionals. He is skilled in both qualitative and quantitative research works	Page 6<br>Lines 155-160
Domain 2: study design		
Theoretical framework		
9. Methodological orientation and theory	Thematic analysis and Grounded theory	Page 8<br>Lines 200
Participant selection		
10. Sampling	Purposive sampling	Page 7<br>Line 175
11. Method of approach	Participants were first made aware of the study by their ward in charges, head of department, health services administrators, in-	Page 7

	service training and research coordinators, human resource managers and medical directors.	Lines 177- 182
12. Sample size	40	Page 7<br><br>Please see Table 1
13. Non-participation	Due to time and workload factors, three participants declined to be interviewed	Page 7<br><br>Lines 182-183
Setting		
14. Setting of data collection	Medical wards, conference rooms and staff office rooms and patient consulting rooms	Page 7-8<br><br>Lines 187-189
15. Presence of non-participants	No	
16. Description of sample	This has been outlined in Table 1 and the section on participants	Page 7<br><br>Lines 168- 173
Data collection		
17. Interview guide	An interview guide and prompts facilitated the interview process. The guide was drafted, pilot tested and consequently revised. See supplementary file 1	Pages 7-8<br><br>Lines 189-192
18. Repeat interviews	No	
19. Audio/visual recording	Yes interviews were audio recorded	Pages 8<br><br>Lines 192-193
20. Field notes	Yes	Page 8<br><br>Line 193
21. Duration	30-60minutes	Page 8<br><br>Lines195-196
22. Data saturation	Yes	Page 7<br><br>Lines 183-184
23. Transcripts returned	Yes but only 13 participants replied with their comments	Page 8<br><br>Line 196-198
Domain 3: analysis and findings		
Data analysis		
24. Number of data coders	1	Page 8<br><br>Lines 203-204
25. Description of the coding tree	No	
26. Derivation of themes	Themes were both identified in advance and emerged from the data	Page 8<br><br>Lines 201-204

27. Software	NVivo version 10.0	Page 8<br>Line 209
28. Participant checking	No	
Reporting		
29. Quotations presented	Yes	Pages 9-17
30. Data and findings consistent	Yes	Pages 9-17
31. Clarity of major themes	Yes	Page 9 and 30
32. Clarity of minor themes	Yes, minor subthemes were presented	Pages 9-17, 30

Supplementary File 2: Characteristics of study hospitals

Hospital	2014 stroke admissions	Hospital bed capacity
TH1	1500	653
TH2	1000	650
TH3	118	500
RH4	409	194
RH5	520	235
RH6	49	200

TH =Tertiary (Teaching) Hospital,

RH= Regional Hospital

Supplementary File 3: Interview Guide

---

a. Let's start with a brief explanation of what you do in this hospital in terms of stroke care? Kindly explain to me what you are expected to do when a stroke patient has been transferred or report to the hospital with a stroke-like symptoms?
b. What different acute stroke care services or treatments are provided for the care of acute stroke patients in this hospital? (Probe for the awareness and use of stroke unit care, aspirin therapy or thrombolytic therapy, etc.)
c. Do you perceive the current acute stroke care services and therapies for stroke patients as helpful in providing care or there are some challenges in using them?
d. How is acute stroke care provided in this hospital? (Probe to understand if the provision of care is guided by clinical guidelines or protocols and if so, what types of guidelines or protocols are used?)
e. Do you find these guidelines helpful in providing care or face some challenges in trying to use them? Where they exist, probe on the following: (their clarity and relevance to stroke clinical care, stroke care professionals familiarity with and confidence in clinical guidelines usage, their attitudes towards clinical guidelines and the perceived barriers of these guidelines in clinical decision-making
f. Now let's discuss the current practical challenges which hinder the delivery of optimal care to stroke patients? Could you elaborate on some of the barriers you face on daily bases? Probe on the following: Guideline factors, health staff level barriers, patient factors, incentives and resources, policy decisions/contexts, national level factors, etc.
g. How do you cope or manage to provide stroke care in the midst of such barriers?
h. What recommendations will you like to make to the hospital authorities on how to improve acute stroke care in the hospital?
i. Is there anything you will like to share, either audio-recorded or off audio recorded in relation to the issues we have just discussed?
j. I will be transcribing the recording and if you don't mind I will be happy to share the interview transcript with you to cross check to be sure what is transcribed reflects your views?
k. Thank you for your time
