# Supplementary material for: Barriers to evidence-based acute stroke care in Ghana: a qualitative study on the perspectives of stroke care professionals
Source: BMJ Open. 2017 Apr 27;7(4):e015385. doi: 10.1136/bmjopen-2016-015385 (PMC5719663; doi:10.1136/bmjopen-2016-015385)
Supplement: Author's manuscript [file bmjopen-2016-015385.draft_revisions.pdf]

Barriers to evidence-based acute stroke care in hospitals: Perspectives of health professionals in Ghana

Journal:	*BMJ Open*
Manuscript ID	bmjopen-2016-015385
Article Type:	Research
Date Submitted by the Author:	01-Dec-2016

Complete List of Authors:	Baatiema, Leonard; University of Ghana, Regional Institute for Population Studies; Australian Catholic University - North Sydney Campus, School of Allied Health (Department of Public Health)<br>De-Graft Aikins, Ama; University of Ghana, Regional Institute for Population Studies<br>Sav, Adem; Australian Catholic University, School of Allied Health (Department of Public Health), Faculty of Health Sciences; Griffith University, Menzies Health Institute<br>Mnatzaganian, George; La Trobe University College of Science Health and Engineering, La Trobe Rural Health School<br>Chan, Carina ; Australian Catholic University, School of Psychology, Faculty of Health Sciences<br>Somerset, Shawn; Australian Catholic University, School of Allied Health (Department of Public Health), Faculty of Health Sciences

**Primary Subject Heading**:	Evidence based practice
Secondary Subject Heading:	Health services research, Health policy, Qualitative research
Keywords:	Barriers, knowledge translation, stroke services, evidence-based practice, Ghana, implementation science

SCHOLARONE™ Manuscripts

Barriers to evidence-based acute stroke care in hospitals: Perspectives of health professionals in Ghana

Leonard Baatiema [1, 2*], Ama De-Graft Aikins[2], Adem Sav[3, 6], George Mnatzaganian[4], Carina K.Y. Chan[5], Shawn Somerset[3]

1. School of Allied Health, Faculty of Health Sciences, Australian Catholic University, Sydney, Australia
2. Regional Institute for Population Studies, University of Ghana, Legon, Accra, Ghana
3. School of Allied Health, Faculty of Health Sciences, Australian Catholic University, Brisbane, Australia
4. College of Science, Health and Engineering, La Trobe Rural Health School, La Trobe University, Melbourne, Australia
5. School of Psychology, Faculty of Health Sciences, Australian Catholic University, Brisbane, Australia
6. Menzies Health Institute, Griffith University, Queensland, Australia

*** Corresponding author**

Leonard Baatiema

Regional Institute for Population Studies,
University of Ghana,
P.O Box LG96
Legon-Accra, Ghana
Email Address: baatiemaleonard@gmail.com

For peer review only - http://bmjopen.bmj.com/site/about/guidelines.xhtml

Abstract

Objective

Despite major advances in acute stroke care interventions, relatively few patients benefit from optimal care due to multiple barriers. Yet current evidence of such barriers is predominantly from high-income countries. This study seeks to understand stroke care professionals' views on the barriers which hinder the provision of optimal acute stroke care in Ghanaian hospital settings.

Design

A qualitative approach using semi-structured interviews. Both thematic and grounded theory approaches were used to analyse and interpret the data through a synthesis of pre-identified and emergent themes.

Setting

A multisite study, conducted in six major referral acute hospital settings (three teaching and three non-teaching regional hospitals) in Ghana.

Participants

A total of 40 participants comprising neurologists, emergency physician specialists, non-specialist medical doctors, nurses, physiotherapists, clinical psychologists and a dietitian.

Results

Four key barriers at the patient, health system, health professionals and broader national health policy levels were identified. Twelve sub-themes of barriers also were identified: financial constraints, delays, socio-cultural or religious practices, discharge against medical advice, denial of stroke, inadequate medical facilities, lack of stroke care protocol, limited staff numbers, inadequate staff development opportunities, poor collaboration, limited knowledge of stroke care interventions and lack of political will. Perceived barriers varied across health professional disciplines and hospitals.

Conclusion

Barriers from low-middle income countries differ substantially from those in high income countries. For evidence-based acute stroke care in low-middle income countries such as Ghana, health policy makers and hospital managers need to consider the contrasts and uniqueness in these barriers in designing quality improvement interventions to optimise patient outcomes.

Keywords: Stroke care, barriers, evidence-based practice, implementation, stroke service, developing countries, Africa

For peer review only

For peer review only - http://bmjopen.bmj.com/site/about/guidelines.xhtml

Strengths and limitations of this study

- Despite the preponderance of evidence on the barriers to uptake of evidence-based stroke care in clinical settings, such evidence is biased towards high income countries. Whilst contributing to this gap, this study represents the first in Ghana to explore, in-depth, the barriers perceived by stroke care professionals to optimum provision of acute care in hospital settings.
- This study focused exclusively on the perspectives of acute stroke care professionals from diverse professional disciplines, expertise, gender, tertiary and non-tertiary hospitals across different geographic settings.
- This study did not focus on barriers to a specific stroke care intervention, as reported extensively in previous works, but rather on barriers across the continuum of stroke care.
- Given the qualitative nature of the study, data interpretation could be subjective and open to bias and thus, caution should be applied in interpretation.

For peer review only

For peer review only - http://bmjopen.bmj.com/site/about/guidelines.xhtml

Background

Recent significant technological advancement in medical practice has increased demands, expectations and pressures on healthcare staff to provide quality and evidence-based care. This is exacerbated by the wide knowledge-clinical practice gap across the world (1), particularly in low-middle income countries (2) where research translation has become an urgent healthcare agenda (3). Empirical evidence in the USA and Europe for example, demonstrates how only about 30% to 50% of patients receive evidence-based interventions in clinical settings (4, 5). It is suggested that translation of an evidenced-based health intervention into routine clinical practice can take up to 17 years (6). The need to identify barriers that underpin the slow uptake of evidence-based care in clinical settings is essential in understanding the extent to which health professionals provide optimal care to patients (5, 7). As a result, theoretical and conceptual attempts have been made to shed light on the factors which affect the current knowledge-practice gap in healthcare settings (5, 8, 9).

Due to the increasing global stroke-related mortality and morbidity (10), the past decades have witnessed a proliferation of evidence-based acute stroke care interventions (11-16). Notwithstanding such advances, uptake of this research in clinical settings remains slow (17, 18), suggesting that only a small proportion of stroke patients receive optimal care. Although the low uptake of these interventions is a global health challenge, evidence suggests the pace of uptake in high income countries exceed that of low-middle income countries (17-19). Numerous barriers have been identified to explain the low uptake of such evidence-based stroke care interventions into routine clinical practice. Some of these barriers include inadequate medical facilities, inadequate knowledge and skill levels of stroke care providers, low awareness of current acute stroke care interventions and the perceived efficacy levels of acute stroke care interventions (20-22). There are also barriers at the patient level which include delays in seeking emergency care due to lack of awareness of early stroke symptoms or financial constraints (23, 24).

For peer review only - http://bmjopen.bmj.com/site/about/guidelines.xhtml

For peer review only

Although research has increased our knowledge about the range of barriers to the uptake of evidence-based stroke care in clinical settings, a more balanced and holistic understanding of such research is needed. Existing research to date only presents a one-sided view, and bias towards high income countries (e.g., Australia and USA) and moreover, is focused primarily on barriers inhibiting uptake of thrombolytic therapy (20, 22, 24, 25). It is unclear if these barriers apply to low-middle income countries as they have different geo-political, socio-economic and health system contexts. An investigation of such barriers is important in low-middle income countries because the global stroke burden is much higher there (10, 26), and yet evidence suggest uptake of evidence-based acute stroke care interventions is relatively lower (18).

This study therefore aimed to identify the views of stroke care professionals on barriers inhibiting the provision of optimal stroke care in Ghanaian hospitals, since such information is non-existent. Greater insights about these barriers and how they differ according to hospital settings and across stroke care professional disciplines are important for developing interventions towards enhancing optimal patient outcomes in Ghana. The findings may also have broader relevance to other resource poor settings.

Methods

Study Design

This study is part of a larger multi-site study to evaluate the provision of evidence-based acute stroke care in acute care in major referral hospitals in Ghana. A qualitative study design using semi-structured interviews was employed to gain a rich and in-depth understanding of the barriers faced by stroke care professionals. The importance of qualitative data to successful translation of best scientific evidence into clinical practice has also been recommended (27). The study design, data collection, analysis and reporting were conducted in accordance with the consolidated criteria for reporting qualitative research (COREQ) (28). See **supporting file 1.**

Settings

The study was conducted in three referral tertiary (teaching) and three regional (non-teaching) hospitals in the southern, middle, and northern belts of Ghana, between November 2015 and April

For peer review only - http://bmjopen.bmj.com/site/about/guidelines.xhtml

2016. The study hospitals are major referral hospitals located in six of the ten administrative regions of Ghana and were chosen to account for the geographic and socio-economic contrasts among the ten administrative regions of the country. The hospital bed capacity for these hospitals is as low as 150 for the regional hospital, whereas the teaching hospitals bed capacity is approximately 653. Overall, the annual stroke admissions for 2014 ranged from 49 for the regional and 1500 stroke cases for the teaching hospitals. The tertiary hospitals are larger referral centres and are well-resourced with diagnostic and therapeutic facilities, whilst the regional hospitals act as major referral points to other hospitals and health centres within their catchment areas.

Participants

Participants comprised key hospital staff primarily involved in directing or providing acute care for stroke patients. To achieve maximum variation in the continuum of care that would reflect a real-life setting, the study recruited nurses, specialist medical doctors (neurologists, emergency physician specialist), non-specialist medical doctors, clinical psychologists, physiotherapists and a dietitian; representing diverse expertise and experience relevant to acute stroke care. Table 1 shows participants' distribution across study sites.

Sampling and Recruitment

Purposive sampling was used to recruit all study participants. Participant recruitment was facilitated by two of the researchers (LB, AA), who were familiar with the study context and had significant qualitative research experience. To commence recruitment and promote the study to eligible participants, meetings were held with hospital administrators, in-service training and research coordinators, department heads and nurses in-charge in the study hospitals. Potential participants were then recommended from these meetings and engagements. Initial contact with prospective participants was made face-to-face or by telephone calls by the first author to identify the date, time and venue for the interviews. Potential participants were identified. Due to time and workload restrictions, three participants declined to participate in the study. The number of participants enrolled into the study was determined by data saturation.

For peer review only - http://bmjopen.bmj.com/site/about/guidelines.xhtml

Data Collection

All interviews were conducted face-to-face in English by LB who had no prior relationship with the interviewees. Data collection was conducted in various venues including: general and emergency wards, consulting rooms, conference rooms, participants' office rooms and physiotherapy departments. The interviews were facilitated by an interview guide (Box 1) developed by the researchers and informed by an extensive literature review on the topic. The interview guide was pilot-tested with three nurses and three medical doctors at non-study sites and adapted to reflect the professional role of the interviewees. With the permission of interviewees, each interview was recorded using a digital voice recorder. Detailed field notes were also taken. The study repeatedly used prompts to facilitate the elicitation of more and clearer information or clarification of certain concepts used by participants. The interviews lasted 45 minutes on average and all recorded interviews were transcribed verbatim by professional transcribers for the final data analysis.

Data Analysis

Thematic data analysis (29, 30), combined with some elements of a grounded theory approach (31), were used to analyse the data. Pre-existing thematic categories based on relevant literature were utilised in the data analysis. The grounded theory, took an inductive approach to ensure all essential emergent themes from the codes not included in the deductive pre-existing coded list of barriers were captured. An initial codebook based on prior codes was developed and subsequently modified with the addition of new emergent themes after a line-by-line reading and re-reading of transcripts by one author (LB). A second author (AA), cross-checked the final coded results with a sample of the transcripts. Using the constant comparison approach (31, 32), a comparative analysis of both emergent and prior themes was conducted between study sites and participants to understand areas of convergence and divergence. NVivo software package version 10.0 (33) was employed to organise, code and identify all data.

Trustworthiness and transferability in the study results were facilitated by the consistent use of the interview guide during the interview process, audio recording of all interviews, professional

For peer review only - http://bmjopen.bmj.com/site/about/guidelines.xhtml

transcription of the interviews and the use of the NVivo software to manage the entire data analysis process. As a measure to further enhance data trustworthiness (34), about one third of the transcripts were shared with a selected participants for cross checking, known as member validation.

Findings

A total of 40 participants took part in the study, approximately six participants per study site. Participants included both males and females of varied professional disciplines, ranks and years of practice in the study sites (**See Table 1**).

Barriers to Acute Stroke Care

Four key themes of barriers to the provision of optimal acute stroke care emerged from the data; patient, hospital or health system, health care providers' and national health policy factors. **Table 2** describes each of these barriers. Embedded in these themes were twelve sub-themes which provided specific and contextualised meaning to the main themes.

Patient factors

Under this category of barriers, five sub-themes were identified: financial constraints, delays, socio-cultural or religious beliefs and practices, discharge against medical advice and denial of stroke.

Financial constraints

In all the study sites and across participants from the various professional disciplines, barriers such as lack of funds to transport patients to the hospital, inability to pay for medical expenses (e.g., CT brain scanning services, laboratory tests and other healthcare associated expenses) were consistently raised. Patients' or their care givers' decision to first seek medical care, organise means of transport to the hospital or pay for medical expenses were often constrained by their level of financial capacity. As a result, access to care was often delayed or deprived. An excerpt from a participant emphasised this:

"poverty and ability to pay for medical cost is the issue over here,…let's say a doctor will request a patient to do a CT scan, do some lab tests …, but the patient just simply cannot afford it, or it takes too

For peer review only - http://bmjopen.bmj.com/site/about/guidelines.xhtml

long for them to gather the money, so for two, three and sometimes five weeks you are treating a patient without a CT scan investigation" (Medical Doctor, ID 9)

Delays

Patient's late arrival to the hospital was commonly cited as another barrier to acute stroke care. Participants suggested the reasons for such delays arose from their lack of awareness of early stroke symptoms and decision to first seek herbal or faith-based care, rather than medical care. Delays were also attributed to financial capacity of the family to seek medical care, especially in instances where the family breadwinner was the stroke victim. Hence, patients with good financial circumstances were more likely to seek early acute medical care compared to those with poor finances.

"…they don't bring the patients early and when they come, they will tell you the condition just started, that they just noticed the symptoms and rushed the patient to the hospital. But you realize that this patient had the stroke for long, not very acute as they described, either they have sought treatments elsewhere or other interventions before arriving here" (Nurse, ID 4)

On the low awareness of early stroke symptoms, one participant noted:

"They don't have knowledge of early stroke symptoms, they are ignorant about stroke symptoms…, Because they don't know what the condition is, patients or families will rather prefer to self-medicate with painkiller or remain at home upon symptoms onset with the hope that the symptoms will disappear" (Medical Doctor, ID 2)

Socio-cultural and religious beliefs

Patient's socio-cultural or religious beliefs and practices emerged as another predominant barrier. Cultural beliefs and practices (e.g. view stroke having a spiritual cause, retribution from their gods and not a condition which can be managed medically) were often very important and likely to influence patient health seeking behaviour. A common practice from such beliefs was patients' desperate attempts to defer medical care for herbal or traditional medical care or make attempts to combine both whilst hospitalised. Some nurses noted such practices or beliefs have often compelled families of patients to abandon medical care in the hospital for alternative care provided by traditional

For peer review only - http://bmjopen.bmj.com/site/about/guidelines.xhtml

or faith healers. For example, all physiotherapists interviewed believed socio-cultural beliefs and practices have limited patient attendance of outpatient care after discharge as most resorted to local herbal treatment options or to prayer camps. A participant had this to say about the religious beliefs on treatment compliance:

".....they become very spiritual once they are diagnosed with a stroke; most now want to focus on their spiritual life instead. You realize that consistently our stroke patients want to talk about God, talking about how lucky they have been, how God has saved them from death" (Clinical Psychologist, ID 1)

Discharge against medical advice

'Discharge against medical advice' was consistently cited by participants and emerged as a key barrier to optimal acute stroke care. This practice was generally perpetuated by two factors; financial capacity to meet medical expenses and families' desire to resort to other forms of care such as traditional herbal medicine, consultation of spiritualists or faith healers. Participants attributed the increased patient and family interest to such alternative forms of care to the vibrant advertisements across the media by traditional herbal medicine practitioners and faith healers. Indeed, promises were made by such individuals to cure stroke and other chronic conditions within a week or two after commencing treatment:

"I recently heard one advertisement which said acute stroke patients should just come here and will be made to walk within a week. So it has gotten to the point where patients easily get misled by these adverts, they find these traditional or faith healers attractive and accessible" (Medical Doctor, ID 11)

According to nurses, such incidences were also linked to the socio-cultural beliefs and practices of the people where health conditions such as stroke, were associated with supernatural or spiritual causes. After being informed about their stroke condition, some stroke patients often insisted to be discharged. Moreover, refusal to heed patients or families' requests for early discharge often resulted in non-compliance to treatment, sudden disappearance of patients or desertion of patients by family members:

For peer review only - http://bmjopen.bmj.com/site/about/guidelines.xhtml

"with the relatives, as soon as they find out that it is a stroke, they start finding ways of transferring the patient to seek herbal medication or to a prayer camp …, so they request for discharge against medical advice and take the patient away" (*Nurse, ID 15)*

Denial

It was also reported that some family members or patients sometimes rejected the diagnosis and dissociated their condition from stroke after being informed about the condition. Participants even acknowledged instances where some family members challenged their professional competence because they felt an incorrect diagnosis was made. The denial of stroke stemmed from the diverse misunderstandings of the illness, with some patients/family members viewing it as an attack or retribution from their gods or spirits for a wrong doing. In such situations, the provision of care was difficult, as some family members were less compliant during treatment:

"I remember one care giver following up to me to inquire whether we were sure the condition of their relative was a stroke, as she believed a wrong diagnosis was documented. Because to her, their relative does not deserve to have a stroke" (Nurse, ID 11)

Hospital or Health system factors

The subthemes of these system factors were shortage of medical facilities/equipment, lack of a stroke specific protocol, inadequate staff numbers and limited staff professional development opportunities.

Shortage of medical facilities

The limited availability of essential medical equipment to facilitate effective provision of acute stroke care was a common feature in study hospitals within the northern belt. There was a shortage of medical facilities such as blood pressure (BP) monitoring apparatus, cardio monitors, suction machines, adjustable hospital beds and inadequate space to facilitate patient care. For example, participants in the only stroke unit in this study believed that the inadequate bed capacity (6-bed capacity) limited admission of many patients to receive optimal care. One participant commented:

For peer review only - http://bmjopen.bmj.com/site/about/guidelines.xhtml

"Unfortunately, you find stroke patients, they come in, no bed, they are sitting on chairs, sitting on the wheel chair or on the bare floors, these are the conditions under which we are expected to provide standard care...."(Medical Doctor, ID 2)

This experience and another comment below exemplified this barrier:

"We have just one oxygen for all the patients in this ward so the nurses are sometimes compelled to use their discretion to wean patients off oxygen to enable another patient benefit if his/her condition is more severe" (Medical Doctor, ID 7)

Additionally, the lack of a stroke unit was a common concern expressed by medical doctors from hospitals in the middle and southern belt, a situation they believed was caused by limited funds allocated by hospitals and a low priority for acute stroke care. A lack of medical equipment and consumables could delay or deprive patients of standard care. Participants also cited instances where some medical doctors acquired personal blood pressure monitoring devices to support patient care because of shortages. Another issue was the absence or frequent malfunction or breakdown of diagnostic services such as CT scanning services, a situation which often delayed care delivery or led to referral of patients to other hospitals. According to some medical doctors, this situation sometimes compelled them to proceed with care delivery without a CT scan investigation to inform treatment options:

"I can say the biggest problem we face is our diagnostic equipment. See the whole of this so called big hospital, we have only one CT scan machine. The machine has been out of service for over 6 to 8 weeks and was only put to use again two weeks ago ...," (Medical Doctor, ID 11)

Lack of a specific protocol for acute stroke care

Most nurses believed the absence of a specific protocol or clinical guideline for acute stroke care was a key barrier:

"....sometimes the cases come and you've forgotten some important procedures because I left the classroom a very long time ago" (Nurse, ID 13)

For peer review only - http://bmjopen.bmj.com/site/about/guidelines.xhtml

One nurse recounted her experience of providing an acute stroke care with much uncertainty because there was no medical doctor or a senior colleague to guide her. This nurse stressed the importance of a clinical protocol, which she argued could facilitate the provision of standardised care even in the absence of a specialist or a medical doctor:

"Most of the stroke cases I have witnessed were rushed in here during late hours, sometimes after mid-night and most times, its only nurses present to attend to the case. So the patient has to wait until a doctor arrives, sometimes the next morning and that is why I think the protocol will at least guide us to safely initiate initial treatment'' (Nurse, ID 18)

Limited staff

Limited staff especially stroke specialists (e.g. neurologists, neurosurgeons and trained stroke nurses) were also a key barrier across the study sites. This issue was more dominant in the non-tertiary regional hospitals and participants in the northern belt of Ghana. Participants, especially nurses, believed the current staff numbers were inadequate to provide optimal acute stroke care (e.g. regular checking of BP levels, sugar levels, regular turning of patients to prevent pressure sores and management of urinal incontinence to minimise risk of urinary tract infections). They expressed frustration about the high work load, which often compromised effective patient care:

"*you could have patients running over 40,…, some are in the wheel chair, some are on the chairs you see over there, some are on the beds, sometimes some are on the stretchers'' (Nurse, ID 5)*

Limited staff professional development opportunities

With the exception of medical doctors, nurses and allied health staff expressed great interest in opportunities for staff professional development; mainly in hands-on training workshops related to stroke clinical care. Although there were policies to support staff develop their current knowledge and skills, such opportunities were very rare. Nurses, for example, emphasised the importance of continuous professional development as current clinical practice was underpinned by what they were taught in schools many years ago. Overall, there was strong opinion on this matter and a lack of

For peer review only - http://bmjopen.bmj.com/site/about/guidelines.xhtml

continuous training opportunities inherently affected the quality of care provided to acute stroke patients:

"we don't have regular workshops…, even if there will ever be such an opportunity, you will only consider attending provided you can afford the cost as this hospital won't support us attend such a workshop" (Nurse, ID 4)

Health care providers' factors

Two main subthemes of barriers were identified at the health care staff level; limited knowledge in acute stroke care and inadequate team collaboration and coordination.

Inadequate knowledge

Lack of knowledge on how to provide appropriate treatment was often discussed, particularly by nurses. Unlike the medical doctors, all the nurses interviewed were unware of thrombolytic therapy. This particular type of therapy was not part of what the medical doctors recommended for acute ischemic stroke care:

"What did you say again? thromboly… what? Not here, I am hearing thrombolysis for the first time. It is not part of our treatment plan for stroke patients in this hospital. How come you say it is one of the key therapies for acute ischemic stroke and I am not aware of it?'' (Nurse, ID 9)

Most nurses also identified insufficient knowledge of certain acute stroke care procedures as a barrier, especially in triaging unconscious stroke patients. They expressed uncertainty about their ability to often proceed with care delivery in the absence of a medical doctor. Although nurses talked about consulting senior colleagues, some level of uncertainty was still noted in proceeding to provide care in the absence of a medical doctor. Despite their clinical training, nurses cited difficulties arising from efforts to respond to unconscious stroke patients and conduct assessment to support an accurate stroke prognosis:

"Sometimes a stroke case arrives unconscious and you start shivering especially when it is in the night and there is no doctor around to respond immediately. I feel very nervous when I realised I am the only senior nurse in the ward to attend to this patient" (Nurse, ID 12)

For peer review only - http://bmjopen.bmj.com/site/about/guidelines.xhtml

For peer review only

Team Collaboration and Communication

According to most nurses and all allied health staff, collaborative work in a multidisciplinary stroke team was inadequate, and an obstacle to effective patient care. Physician driven stroke care without adequate involvement of other staff, was frequently discussed.

"I don't even think we have a working team here, it is more of a doctor giving instructions…, giving instructions to nurses, though nurses are there with the patients 24 hours, the medical doctors just come to see their patients and then disappear. Is that what you call teamwork?" (Nurse, ID 14)

Allied health staff expressed a sense of marginalisation and disconnectedness, especially in the early stages of care. A dietitian for example cited instances where medical teams (doctors and nurses) often discharge patients without his view on dietary plans at discharge. Some physiotherapists expressed similar concerns of limited involvement which in their view inhibited their ability to develop initial rapport with patients or the opportunity to educate patients about the importance of self-care practices, following discharge:

"There was an occasion my rounds coincided with the medical team's rounds; I quickly joined them. I made a suggestion on a particular patient we were attending to but this was brushed off and the medical doctor behaved as if I was trying to direct him what to do or take over his job" (Physiotherapist, ID 3)

National policy context factors

Participants identified one key barrier under this theme; lack of political will for acute stroke care.

Lack of Political Will

The lack of national level support and political will for acute stroke care was consistently cited as a broad level barrier, particularly by medical doctors. They expressed strong views on this issue, attributing it to the increasing out-of-pocket medical expenses for patients. Despite the existence of the national health insurance policy which was supposed to replace the practice of 'cash and carry', a lack of political will for the scheme has gradually introduced the policy of upfront payments by patients prior to acute care in most hospitals in Ghana presently. They believed this has negatively

For peer review only - http://bmjopen.bmj.com/site/about/guidelines.xhtml

affected patients' access to care (not only stroke patients) because of their inability to pay for medical expenses. The limited coverage of the national health insurance scheme on chronic care, such as stroke, was also stated as a key barrier. Patients' experienced difficulties paying for stroke-related medical costs (e.g., CT brain scans and other laboratory tests) that were not covered by the national health insurance scheme. Overall, there was a sense of powerlessness about national level neglect for acute stroke care. Consequently, this resulted in staff dissatisfaction and a lack of motivation to provide effective care.

"The problems we face in our current health sector has very little to do with health professionals' reluctance to provide standard care. It is the health system! …., we are under a system where every medication is expensive for the ordinary Ghanaian to afford and yet, we make the patients to believe the national health insurance policy covers everything. Now almost every medication has to be paid for by the patient and if they can't afford what we recommend as best treatment option for their condition, we provide the alternatives which may not be very effective" (Medical Doctor, ID 11).

Discussion

Summary of main findings

This study provides in-depth insights of the barriers to the delivery of optimal acute stroke care in Ghana, a largely neglected low-middle income country in Africa. The findings suggest that although the barriers identified share some commonality with those reported in previous studies in high income countries, some barriers are unique to optimal stroke care in low-middle income settings such as Ghana. For example, contrary to barriers found in high income countries, the issue of discharge against medical advice and the role of socio-cultural and religious beliefs or practices of stroke patients and their families characterise the present study. Whilst illuminating the reasons influencing the provision of optimal acute stroke care in a typical resource poor setting, the findings further unravel barriers peculiar to different stroke care professionals and hospital settings where much policy attention is required for effective and timely translation of evidence-based stroke care intervention into routine clinical practice.

For peer review only - http://bmjopen.bmj.com/site/about/guidelines.xhtml

Comparison with previous literature

As found in this study, stroke care in high income countries with modern resources has consistently reported barriers corresponding to patient, health system/hospital, health staff and the national level factors (20, 22, 35). Highlighting patient level barriers as the most predominant of all barriers identified in our study, a previous study corroborated this by reporting 91% of participants viewed prehospital delay at the patient level as the most dominant barrier to providing thrombolytic therapy (24). An earlier study in Ghana (36) found that only 40% (277/693) could correctly identify stroke symptoms, also reinforces the importance of our finding that participants identified patient delays to seek care due to low awareness of stroke symptoms.

Although our findings on the importance of patient-level barriers to optimal stroke care, are in line with previous research, the explanation and the circumstances in which some patient level factors acted as barriers to optimal acute stroke care were somewhat different in this study. For example, socio-cultural or religious beliefs and practices were perceived to underpin health seeking behaviours of stroke patients and their families in Ghana. Although this is inconsistent with the literature on barriers to acute stroke care in high income countries, our findings corroborate with research on other chronic diseases and health seeking behaviour in Ghana (37-39). This underscores the influence such beliefs and practices to health seeking behaviours of patients and families in Ghana. Evidence within the African contexts suggest patient access to traditional and faith healers as complimentary avenues of care is due to the easy access, lower cost and cultural legitimacy of such alternatives (40, 41).

In addition, patient discharge against medical advice was also a key barrier affecting optimal clinical care. This finding is also largely inconsistent with published barriers to acute stroke care from high income countries. Despite the limited popularity of such barriers in previous studies, this has been well articulated in other health contexts and conditions with conclusive arguments of the practice being a drawback and an obstacle to provision of adequate and quality health care (42-45). Clearly, this issue requires further investigation in Ghana, and possibly other low-middle income countries. Larger studies would clarify the extent to which this can affect standard acute stroke care.

For peer review only - http://bmjopen.bmj.com/site/about/guidelines.xhtml

Other important barriers from this study were related to the health system such as limited stroke care specialists, increased workload for staff, inadequate medical facilities, lack of protocols and unavailability or limited access to CT brain scans. The importance of this set of barriers has been reported previously (20, 46, 47), highlighting the extent to which they affect provision of optimal patient care. For example, one study found that 71% of participants identified lack of protocols, care paths and opportunities for staff professional education as important barriers to the provision of optimal acute stroke care (24). Comparable to our study, a Swedish study identified low staffing levels as a major barrier to optimal stroke care (22). Despite these studies being conducted in high income countries, their corroboration with the present study reinforces the importance of hospital/health system level barriers to the uptake of evidence-based practice.

The issue of limited collaboration or involvement of allied health staff and other providers in the provision of care is also worthy of attention. Multidisciplinary and coordinated care remains a central component in contemporary evidence-based practice for acute stroke care (48, 49). As a result, inadequate involvement of these staff is a significant issue since participants noted that their limited involvement is detrimental to optimal patient care. Evidence from existing scholarship on such barriers has been previously reported (50-52), thus stressing the need to consider interventions to improve collaboration among stroke care professionals in acute stroke care.

Nurses' knowledge of acute stroke care interventions such as thrombolytic therapy was also identified as a barrier, consistent with previous (20, 24, 35). This issue has also been identified in an Australian study where 50% of nurses reported having limited knowledge of thrombolytic therapy (24). Such findings highlight the importance of this issue to optimal stroke care in both low-middle-income and high-income countries.

Finally, another barrier identified in the present study relates to the low level of political will for optimal acute stroke care. This barrier seems unique to Ghana and reflects the absence of national stroke clinical guidelines, a national framework for quality improvement interventions for stroke and limited coverage of the national health insurance scheme to cover patients' medical expenses. Whilst this finding corroborates with previous studies asserting the limited prioritization of acute stroke care

For peer review only - http://bmjopen.bmj.com/site/about/guidelines.xhtml

by health policy makers in resource poor settings (53-56), this could also likely be symptomatic of the current limited global health funding for stroke and other non-communicable diseases compared to communicable diseases (57).

Implications for future research, policy and clinical practice

The present findings have several important implications for the provision of evidence-based acute stroke care in Ghana. Firstly, patient financial constraints appear to be a key barrier to optimal care and needs urgent attention. It is apparent that patients and family members struggle with the financial costs of stroke treatment and strategies are needed to overcome this burden. The current Ghana National Health Insurance Policy offers limited financial risk protection for stroke care. This epitomises the fragile nature of health care systems in low-middle income countries, which require significant structural and policy reforms to minimise the current high cost of treatment for chronic diseases such as stroke. If left unaddressed, the consequent increased incidence of late arrival or refusal to seek care, limited treatment options and patient discontinuation of treatment will negatively affect optimal patient outcomes. Interestingly, the cost of treatment and healthcare seems not be unique to only low-middle income country such as Ghana, but is also widely reported in high-middle income countries, such as Australia (58). Clearly, interventions to reduce the cost of treatment for chronic health conditions are needed, not only in Ghana, but also in other high-income countries.

Secondly, the evidence of socio-cultural or religious beliefs and practices as a barrier to optimal care also deserves attention, particularly since this has received little attention in the current literature on barriers to acute stroke care. More research in other settings may be useful to unravel the extent to which such practices influence provision of optimal care. In addition, interventions to increase the health literacy regarding stroke risk factors, increase public awareness of early stroke symptoms and the need to seek early medical care are critical. This level of public awareness and education campaigns have been implemented in high income countries such as UK (59, 60), Australia (61, 62), Canada (63) and Germany (64). Hence Ghana and other low-middle-income countries can clearly draw lessons from such public awareness campaigns on early recognition of stroke symptoms to minimise patient delays to seek care. Such interventions however would need to be adapted to suit the

For peer review only - http://bmjopen.bmj.com/site/about/guidelines.xhtml

particular country and health context. They should be mainstreamed in the healthcare systems of such countries through collaboration with the public and private sectors to optimise the impact.

Importantly, the finding of patient discharge against medical advice has revived debates about the place of patients within the current evidence-based medicine paradigm where patient's needs and preferences are essential (65). Whilst more research on the implications of this issue is required, institutional measures exploring safe and appropriate times and conditions under which such requests could be granted should be identified. More importantly, strategies need to be adopted to ensure that requests by patients and families to be discharged against medical advice be counterbalanced with tailored communication to improve awareness of the risks and benefits. The roles of clinical psychologists and nurses can be pivotal in such communication.

The limited collaboration and poor communication among stroke care professionals also warrants attention. Highlighted as an imperative in providing optimal acute stroke care by previous research (12, 51, 52), the finding in this study further emphasises the need to explore effective ways to build collaborative working environments. Structural policy reforms are needed to ensure equal respect for individual professional experiences, identity, autonomy and responsibilities. Staff professional development has also been highlighted as critical to stroke care quality improvement (66) and overall health outcomes (67), and as such, efforts to provide staff educational and professional development opportunities in stroke care could be useful in the Ghanaian setting.

Given that health system and hospital level factors were observed as important to stroke care, strengthening health systems through the provision of adequate and effective acute stroke care services is essential. For example, to address the issue of limited staff numbers, an immediate short term measure by health managers and policy makers would be to consider task shifting approaches, as has been trialled in Nigeria. This Nigerian study (68) showed improved knowledge of non-neurologists in acute stroke care, thus potentially translating into improved patient outcomes.

Finally, to increase implementation of evidence-based acute stroke care, there is the need for increased policy commitment for optimal acute stroke care through increased allocation of resources to hospitals in the form of infrastructural support, a comprehensive coverage of the current national

For peer review only - http://bmjopen.bmj.com/site/about/guidelines.xhtml

health insurance policy to include CT brain scan services and medical expenses for chronic care, staff professional development opportunities, and development of a stroke specific clinical guideline are urgently needed. All the aforementioned factors should be considered in developing acute stoke care interventions in Ghana and indeed other low-middle income countries as potential pathways to ensuring the current global evidence-based acute stroke interventions, especially in low-middle income countries are routinely practised in clinical settings for positive patient outcomes.

Limitations and Strengths

As a limitation, this study reported results from limited participants whose views may not be reflective of the wider health staff responsible for acute stroke care in Ghana. Nonetheless, this was conducted in six major referral hospitals in six of the ten administrative regions of Ghana and so the findings may be applicable to other stroke care professionals. Future studies should target a larger and more representative study sample to include district and municipal hospitals. Further, given the qualitative nature of the study, data interpretation could be subjective and open to bias and thus, caution should be applied in interpretation. However, the use of participant cross checking and validation of interview transcripts, and the consistent use of the interview guide during the interview process enhanced the study validity and reliability.

Notwithstanding these limitations, this study also has several strengths. First, to our knowledge, this study represents the first in Ghana to explore, in-depth, the barriers perceived by stroke care professionals to optimum provision of acute care in hospital settings. Another key strength is its exclusive focus on the perspectives of acute stroke care professionals from diverse professional disciplines, expertise, gender, tertiary and non-tertiary hospitals across different geographic settings. Using qualitative design, the findings provide contextually rich information of such barriers which would have been more difficult to unravel quantitatively. The study findings provide some new insights of other factors which have been less recognised in previous literature on the barriers to evidence-based acute stroke care. Added to this, this study did not focus on barriers to a specific stroke care intervention, as reported extensively in previous works, but rather on barriers across the continuum of stroke care.

For peer review only - http://bmjopen.bmj.com/site/about/guidelines.xhtml

For peer review only

Conclusion

Overall, the views on barriers to optimal stroke care varied significantly based on specific professional discipline and study sites. Although most of the barriers were largely consistent with previous studies in high income countries, the study unravelled some unique barriers which extend the body of literature on barriers to acute stroke care. Importantly, barriers in low-middle income countries showed important differences to those from high income countries. Greater political will for acute stroke care in terms of increased coverage of the national health insurance scheme, increased resource allocation, recruitment and training of an expanded stroke health workforce could improve uptake of evidence-based acute stroke care interventions. The information provided in this paper is potentially important to health managers, policy makers, patients, grant managers or holders and other health stakeholders as it presents various reasons why delivery of acute stroke care in clinical setting may be far from optimum. To this end, to translate current evidence-based acute stroke interventions for optimal patient outcomes in Ghana and potentially in other resource poor settings, a clear-cut understanding of these barriers to inform policy formulation, quality improvement and staff professional development is critical.

Acknowledgement

The authors gratefully acknowledge the support received from the staff of the study hospitals during field data collection. The contribution of Dr Michael Otim, Associate Professor Liz Mclnnes and Professor Sandy Middleton to the study conception are greatly acknowledged.

Authors Contribution

Study design and instrumentation process: LB, AA, GM, SS. Participants recruitment: LB, AA. Data collection, analysis, interpretation and writing of first manuscript: LB. Contribution to study interpretation and critical review of the manuscript: LB, AA, AS, GM, CKYC and SS. All authors have made substantial contribution to the writing of this manuscript for critical intellectual content. All authors have reviewed and approved the final version for submission.

Funding

For peer review only - http://bmjopen.bmj.com/site/about/guidelines.xhtml

The first author (LB) is a PhD Candidate funded under the University International Students Scholarship programme. LB also received funding support from the University Faculty of Health Science Higher Degree Research (HDR) Student Support Scheme during his candidature. However, these funding bodies did not play a role in the study design, data collection and analysis, results interpretation, writing of the entire manuscript and the decision to submit the manuscript to this journal.

Competing Interest

The authors declare no competing interests

Ethics

This study received ethical approval from the University Human Research Ethics Committee (2015-154H), the Ghana Health Service Ethical Review Committee on Research Involving Human Subjects (GHS-ERC: 11/07/15) and the Committee on Human Research Publications and Ethics of the School of Medical Sciences of the Kwame Nkrumah University of Science and Technology and the Komfo Anokye Teaching Hospital (CHRPE/AP/141/16). The Institutional Review Board of the 37 Military Hospital (37MH-IRB IPN 035/2015) also granted clearance for the conduct of this study.

Data sharing statement: No additional data are available

Reference

1. World Health Organization, editor Bridging the "Know–Do" Gap. Meeting on knowledge translation in global health; 2006: Gene-va: WHO.
2. Hutin YJ, Hauri AM, Armstrong GL. Use of injections in healthcare settings worldwide, 2000: literature review and regional estimates. Bmj. 2003;327(7423):1075.
3. Chan CK, Oldenburg B, Viswanath K. Advancing the Science of Dissemination and Implementation in Behavioral Medicine: Evidence and Progress. International journal of behavioral medicine. 2015;22(3):277-82.
4. Schuster MA, McGlynn EA, Brook RH. How good is the quality of health care in the United States? Milbank Quarterly. 2005;83(4):843-95.
5. Grol R, Wensing M, Eccles M, Davis D. Improving patient care: the implementation of change in health care: John Wiley & Sons; 2013.
6. Morris ZS, Wooding S, Grant J. The answer is 17 years, what is the question: understanding time lags in translational research. Journal of the Royal Society of Medicine. 2011;104(12):510-20.

For peer review only - http://bmjopen.bmj.com/site/about/guidelines.xhtml

7. Hamilton S, McLaren S, Mulhall A. Assessing organisational readiness for change: use of diagnostic analysis prior to the implementation of a multidisciplinary assessment for acute stroke care. Implementation Science. 2007;2(1):1.
8. Straus S, Tetroe J, Graham ID. Knowledge translation in health care: moving from evidence to practice: John Wiley & Sons; 2013.
9. SURE Collaboration. SURE Guides for Preparing and Using Evidence-Based Policy Briefs: 5 Identifying and addressing barriers to implementing policy options. 2011.
10. Mensah GA, Norrving B, Feigin VL. The global burden of stroke. Neuroepidemiology. 2015;45(3):143-5.
11. Sandercock P, Counsell C, Tseng M, Cecconi E. Oral antiplatelet therapy for acute ischaemic stroke (Review). Cochrane Database Syst Rev. 2014(3).
12. Trialists'Collaboration SU. Organised inpatient (stroke unit) care for stroke. Cochrane Database Syst Rev. 2013;9.
13. Wardlaw JM, Murray V, Berge E, del Zoppo GJ. Thrombolysis for acute ischaemic stroke. The Cochrane Library. 2014.
14. Vahedi K, Hofmeijer J, Juettler E, Vicaut E, George B, Algra A, et al. Early decompressive surgery in malignant infarction of the middle cerebral artery: a pooled analysis of three randomised controlled trials. The Lancet Neurology. 2007;6(3):215-22.
15. Donnan GA, Davis SM, Parsons MW, Ma H, Dewey HM, Howells DW. How to make better use of thrombolytic therapy in acute ischemic stroke. Nature Reviews Neurology. 2011;7(7):400-9.
16. Donnan GA, Fisher M, Macleod M, Davis SM. Stroke. The Lancet. 2008;371(9624):1612-23.
17. Langhorne P, de Villiers L, Pandian JD. Applicability of stroke-unit care to low-income and middle-income countries. The Lancet Neurology. 2012;11(4):341-8.
18. Berkowitz AL, Mittal MK, McLane HC, Shen GC, Muralidharan R, Lyons JL, et al. Worldwide reported use of IV tissue plasminogen activator for acute ischemic stroke. International Journal of Stroke. 2014;9(3):349-55.
19. Rhoda A, Smith M, Putman K, Mpofu R, DeWeerdt W, DeWit L. Motor and functional recovery after stroke: a comparison between rehabilitation settings in a developed versus a developing country. BMC health services research. 2014;14(1):82.
20. Meurer WJ, Majersik JJ, Frederiksen SM, Kade AM, Sandretto AM, Scott PA. Provider perceptions of barriers to the emergency use of tPA for acute ischemic stroke: a qualitative study. BMC emergency medicine. 2011;11(1):5.
21. Purvis T, Moss K, Denisenko S, Bladin C, Cadilhac DA. Implementation of evidence-based stroke care: enablers, barriers, and the role of facilitators. Journal of multidisciplinary healthcare. 2014;7:389.
22. Stecksén A, Lundman B, Eriksson M, Glader E-L, Asplund K. Implementing Thrombolytic Guidelines in Stroke Care Perceived Facilitators and Barriers. Qualitative health research. 2013:1049732313514137.
23. Hargis M, Shah JN, Mazabob J, Rao CV, Suarez JI, Bershad EM. Barriers to administering intravenous tissue plasminogen activator (tPA) for acute ischemic stroke in the emergency department: A cross-sectional survey of stroke centers. Clinical Neurology and Neurosurgery. 2015;135:79-84.
24. Williams JM, Jude MR, Levi CR. Recombinant tissue plasminogen activator (rt-PA) utilisation by rural clinicians in acute ischaemic stroke: A survey of barriers and enablers. Australian Journal of Rural Health. 2013;21(5):262-7.

For peer review only - http://bmjopen.bmj.com/site/about/guidelines.xhtml

25. Grady A, Bryant J, Carey M, Paul C, Sanson-Fisher R. Enablers of the Implementation of Tissue Plasminogen Activator in Acute Stroke Care: A Cross-Sectional Survey. PloS one. 2014;9(12):e114778.
26. Feigin VL, Lawes CM, Bennett DA, Barker-Collo SL, Parag V. Worldwide stroke incidence and early case fatality reported in 56 population-based studies: a systematic review. The Lancet Neurology. 2009;8(4):355-69.
27. Greenhalgh T, Howick J, Maskrey N. Evidence based medicine: a movement in crisis? 2014.
28. Tong A, Sainsbury P, Craig J. Consolidated criteria for reporting qualitative research (COREQ): a 32-item checklist for interviews and focus groups. International Journal for Quality in Health Care. 2007;19(6):349-57.
29. Braun V, Clarke V. Using thematic analysis in psychology. Qualitative research in psychology. 2006;3(2):77-101.
30. Bryman A. Social research methods: Oxford university press; 2012.
31. Strauss A, Corbin J. Basics of qualitative research: Techniques and procedures for developing grounded theory: Sage Publications, Inc; 1998.
32. Bradley EH, Curry LA, Devers KJ. Qualitative data analysis for health services research: developing taxonomy, themes, and theory. Health services research. 2007;42(4):1758-72.
33. QSR International. NVivo Version 10.0 [Computer software] 2015. Available from: http://www.qsrinternational.com/.
34. Mays N, Pope C. Assessing quality in qualitative research. British medical journal. 2000;320(7226):50.
35. Donnellan C, Sweetman S, Shelley E. Health professionals' adherence to stroke clinical guidelines: a review of the literature. Health policy. 2013;111(3):245-63.
36. Donkor E, Owolabi M, Bampoh P, Aspelund T, Gudnason V. Community awareness of stroke in Accra, Ghana. BMC Public Health. 2014;14(1):196.
37. Aikins Ad-G. Healer shopping in Africa: new evidence from rural-urban qualitative study of Ghanaian diabetes experiences. Bmj. 2005;331(7519):737.
38. Teye A. Psychosocial Well-Being of Individuals Living with Chronic Non-Communicable Diseases in Urban Poor Communities of Ghana: University of Ghana; 2013.
39. de-Graft Aikins A, Pitchforth E, Allotey P, Ogedegbe G, Agyemang C. Culture, ethnicity and chronic conditions: reframing concepts and methods for research, interventions and policy in low-and middle-income countries. Ethnicity & health. 2012;17(6):551-61.
40. Awah PK, Unwin N, Phillimore P. Cure or control: complying with biomedical regime of diabetes in Cameroon. BMC Health Services Research. 2008;8(1):1.
41. Marshall IJ, Wolfe CD, McKevitt C. Lay perspectives on hypertension and drug adherence: systematic review of qualitative research. 2012.
42. Choi M, Kim H, Qian H, Palepu A. Readmission rates of patients discharged against medical advice: a matched cohort study. PloS one. 2011;6(9):e24459.
43. Glasgow JM, Vaughn-Sarrazin M, Kaboli PJ. Leaving against medical advice (AMA): risk of 30-day mortality and hospital readmission. Journal of general internal medicine. 2010;25(9):926-9.
44. Southern WN, Nahvi S, Arnsten JH. Increased risk of mortality and readmission among patients discharged against medical advice. The American journal of medicine. 2012;125(6):594-602.
45. Garland A, Ramsey CD, Fransoo R, Olafson K, Chateau D, Yogendran M, et al. Rates of readmission and death associated with leaving hospital against medical advice: a population-based study. Canadian Medical Association Journal. 2013;185(14):1207-14.

For peer review only - http://bmjopen.bmj.com/site/about/guidelines.xhtml

46. Hamilton S, McLaren S, Mulhall A. Assessing organisational readiness for change: use of diagnostic analysis prior to the implementation of a multidisciplinary assessment for acute stroke care. Implementation Science. 2007;2.

47. O'Rourke F, Chan DKY, Chan DL, Ding XM. Preferences, barriers and facilitators for establishing comprehensive stroke units: a multidisciplinary survey. Australian Health Review. 2013;37(3):318-23.

48. Langhorne P, Pollock A. What are the components of effective stroke unit care? Age and Ageing. 2002;31(5):365-71.

49. Trialists'Collaboration SU. Organised inpatient (stroke unit) care for stroke. Cochrane database of systematic reviews. 2013(4).

50. Bayley MT, Hurdowar A, Richards CL, Korner-Bitensky N, Wood-Dauphinee S, Eng JJ, et al. Barriers to implementation of stroke rehabilitation evidence: findings from a multi-site pilot project. Disability and rehabilitation. 2012;34(19):1633-8.

51. Gibbon B, Watkins C, Barer D, Waters K, Davies S, Lightbody L, et al. Can staff attitudes to team working in stroke care be improved? Journal of advanced nursing. 2002;40(1):105-11.

52. Baxter SK, Brumfitt SM. Professional differences in interprofessional working. Journal of Interprofessional Care. 2008;22(3):239-51.

53. Cadilhac D, Lalor E, Pearce D, Levi C, Donnan G. Access to stroke care units in Australian public hospitals: facts and temporal progress. Internal Medicine Journal. 2006;36(11):700-4.

54. Fuster V, Voute J, Hunn M, Smith SC. Low Priority of Cardiovascular and Chronic Diseases on the Global Health Agenda A Cause for Concern. Circulation. 2007;116(17):1966-70.

55. Rudd A, Hoffman A, Down C, Pearson M, Lowe D. Access to stroke care in England, Wales and Northern Ireland: the effect of age, gender and weekend admission. Age and Ageing. 2007;36(3):247-55.

56. Aikins Ad-G, Boynton P, Atanga LL. Review Developing effective chronic disease interventions in Africa: insights from Ghana and Cameroon. 2010.

57. Institute for Health Metrics and Evaluation. Financing Global Health 2015 :Development assistance steady on the path to new Global Goals. Seattle, WA: Institute for Health Metrics and Evaluation, 2016.

58. Sav A, Whitty JA, McMillan SS, Kendall E, Kelly F, King MA, et al. Treatment Burden and Chronic Illness: Who is at Most Risk? The Patient-Patient-Centered Outcomes Research. 2016:1-11.

59. Dombrowski SU, Mackintosh JE, Sniehotta FF, Araujo-Soares V, Rodgers H, Thomson RG, et al. The impact of the UK 'Act FAST'stroke awareness campaign: content analysis of patients, witness and primary care clinicians' perceptions. BMC public health. 2013;13(1):915.

60. Flynn D, Ford GA, Rodgers H, Price C, Steen N, Thomson RG. A time series evaluation of the FAST national stroke awareness campaign in England. PloS one. 2014;9(8):e104289.

61. Bray JE, Mosley I, Bailey M, Barger B, Bladin C. Stroke public awareness campaigns have increased ambulance dispatches for stroke in Melbourne, Australia. Stroke. 2011;42(8):2154-7.

62. Bray JE, Johnson R, Trobbiani K, Mosley I, Lalor E, Cadilhac D. Australian public's awareness of stroke warning signs improves after national multimedia campaigns. Stroke. 2013;44(12):3540-3.

63. Hodgson C, Lindsay P, Rubini F. Can mass media influence emergency department visits for stroke? Stroke. 2007;38(7):2115-22.

For peer review only - http://bmjopen.bmj.com/site/about/guidelines.xhtml

64. Worthmann H, Schwartz A, Heidenreich F, Sindern E, Lorenz R, Adams HA, et al. Educational campaign on stroke in an urban population in Northern Germany: influence on public stroke awareness and knowledge. International Journal of Stroke. 2013;8(5):286-92.
65. Dopson S, Locock L, Gabbay J, Ferlie E, Fitzgerald L. Evidence-based medicine and the implementation gap. Health:. 2003;7(3):311-30.
66. Morgenstern LB, Bartholomew LK, Grotta JC, Staub L, King M, Chan W. Sustained benefit of a community and professional intervention to increase acute stroke therapy. Archives of Internal Medicine. 2003;163(18):2198-202.
67. Greiner AC, Knebel E. Health professions education: A bridge to quality: National Academies Press; 2003.
68. Akinyemi RO, Owolabi MO, Adebayo PB, Akinyemi JO, Otubogun FM, Uvere E, et al. Task-shifting training improves stroke knowledge among Nigerian non-neurologist health workers. Journal of the Neurological Sciences. 2015;359(1-2):112-6.

For peer review only

Table 1: Distribution of interview participants and study hospitals

Participants	Tertiary/Teaching Hospitals	Regional Hospitals	Total
Nurses	11	9	20
Medical Doctors/Physicians	6	6	12
Clinical Psychologist	1	1	2

For peer review only - http://bmjopen.bmj.com/site/about/guidelines.xhtml

Physiotherapist	2	3	5
Dietitian	0	1	1
**Total**	**20**	**20**	**40**

For peer review only

Table 2: Themes and definitions

Coding Categories	Definition of Barriers
Patient level	Includes factors, such as late arrival or low awareness of stroke symptoms, denial of stroke, financial capacity, socio-cultural practices or beliefs inhibiting access or adherence to optimal acute stroke care.
Hospital or health system level	Relates to a lack of inadequate medical facilities or equipment, staff numbers, protocols, management support,

For peer review only - http://bmjopen.bmj.com/site/about/guidelines.xhtml

	supporting policies, organisational context or norms which support implementation of standard care and availability of staff professional development opportunities to support the provision of standard care
Stroke care professionals	Describes acute stroke care providers' level of team support, communication or collaborations which affect the provision of care. Also includes competence, skill, knowledge, awareness, familiarity or agreement to specific treatments, their values, motivations or attitudes towards particular treatments or intervention
National /state health policy context	Relates to the level of political will for acute stroke care in the form of national stroke policies, limited allocation of resources for acute stroke care, reimbursement of funds to hospitals, national health policies to support stroke patients access to optimal care and the lack of any regulatory frameworks or policies to support stroke care.

For peer review only

For peer review only - http://bmjopen.bmj.com/site/about/guidelines.xhtml

Supplementary File 2: Interview Guide

a. Let's start with a brief explanation of what you do in this hospital in terms of stroke care? Kindly explain to me what you are expected to do when a stroke patient has been transferred or report to the hospital with a stroke-like symptoms?
b. What different acute stroke care services or treatments are provided for the care of acute stroke patients in this hospital? (Probe for the awareness and use of stroke unit care, aspirin therapy or thrombolytic therapy, etc.)
c. Do you perceive the current acute stroke care services and therapies for stroke patients as helpful in providing care or there are some challenges in using them?
d. Is the provision of care guided by clinical guidelines or protocols? If so, what times of guidelines of protocols are used in this hospital?
e. Do you find these guidelines helpful in providing care or face some challenges in trying to use them? Where they exist, probe on the following: (their clarity and relevance to stroke clinical care, stroke care professionals familiarity with and confidence in clinical guidelines usage, their attitudes towards clinical guidelines and the perceived barriers of these guidelines in clinical decision-making
f. Now let's discuss the current practical challenges which hinder the delivery of optimal care to stroke patients? Could you elaborate on some of the barriers you face on daily bases? Probe on the following: Guideline factors, health staff level barriers, patient factors, incentives and resources, policy decisions/contexts, national level factors, etc.
g. How do you cope or manage to provide stroke care in the midst of such barriers?
h. What recommendations will you like to make to the hospital authorities on how to improve acute stroke care in the hospital?
i. Is there anything you will like to share, either audio-recorded or off audio recorded in relation to the issues we have just discussed?
j. I will be transcribing the recording and if you don't mind I will be happy to share the interview transcript with you to cross check to be sure what is transcribed reflects your views?
k. Thank you for your time

For peer review only - http://bmjopen.bmj.com/site/about/guidelines.xhtml

Supporting File 1: Consolidated criteria for reporting qualitative studies (COREQ): 32-item checklist

Item	Guide questions/description
**Domain 1: Research team and reflexivity**	
Personal Characteristics	
1. Interviewer/facilitator	LB and AA facilitated the recruitment process. LB conducted the interviews
2. Credentials	LB (MSc), AA (PhD), GM (PhD), AS(PhD), CKYC (PhD) SS (PhD)
3. Occupation	LB (Public Health PhD Candidate), AA (Professor of Social Psychology and Population Studies), GM (Epidemiologist and Public Health Lecturer), AS (Health Services Researcher and Public Health Lecturer), CKYC (Senior Lecturer in Health Psychologist , Knowledge dissemination and implementation researcher) SS (Associate Professor of Public Health)
4. Gender	Four researchers were males and two were females
5. Experience and training	LB AA, and AS have extensive experience in conducting qualitative research studies. GM, CKYC, SS have relevant training and knowledge in qualitative research studies.
Relationship with participants	
6. Relationship established	No
7. Participant knowledge of the interviewer	LB and AA are familiar with the study contexts through previous works so it is possible some participants have met or are aware of their works.
8. Interviewer characteristics	All interviewers are interested in research on implementation science and quality improvement interventions for stroke care health professionals.
**Domain 2: study design**	
Theoretical framework	
9. Methodological orientation and Theory	Thematic analysis and Grounded theory
Participant selection	
10. Sampling	Purposive sampling
11. Method of approach	Participants were first made aware of the study by their ward in charges, head of department, health services

For peer review only - http://bmjopen.bmj.com/site/about/guidelines.xhtml

	administrators, in-service training and research coordinators, human resource managers and medical directors.
12. Sample size	40
13. Non-participation	Due to time and workload factors, three participants declined to be interviewed
Setting	
14. Setting of data collection	Medical wards, conference rooms and staff office rooms and patient consulting rooms
15. Presence of non-participants	No
16. Description of sample	This has been outlined in Table 1 and the section on participants
Data collection	
17. Interview guide	An interview guide and prompts facilitated the interview process. The guide was drafted, pilot tested and consequently revised.
18. Repeat interviews	No
19. Audio/visual recording	40 interviews were audio recorded
20. Field notes	Yes
21. Duration	30-60minutes
22. Data saturation	Yes
23. Transcripts returned	Yes but only 13 participants replied with their comments
**Domain 3: analysis and findings**	
Data analysis	
24. Number of data coders	1
25. Description of the coding tree	No
26. Derivation of themes	Themes were both identified in advance and emerged from the data
27. Software	NVivo version 10.0
28. Participant checking	Yes
Reporting	
29. Quotations presented	Yes
30. Data and findings consistent	Yes
31. Clarity of major themes	Yes
32. Clarity of minor themes	Yes, minor subthemes were presented

For peer review only - http://bmjopen.bmj.com/site/about/guidelines.xhtml

For peer review only

BMJ Open

Barriers to evidence-based acute stroke care in Ghana: A qualitative study on the perspectives of stroke care professionals

Journal:	*BMJ Open*
Manuscript ID	bmjopen-2016-015385.R1
Article Type:	Research
Date Submitted by the Author:	22-Feb-2017
Complete List of Authors:	Baatiema, Leonard; University of Ghana, Regional Institute for Population Studies; Australian Catholic University - North Sydney Campus, School of Allied Health (Department of Public Health)<br>De-Graft Aikins, Ama; University of Ghana, Regional Institute for Population Studies<br>Sav, Adem; Australian Catholic University, School of Allied Health (Department of Public Health), Faculty of Health Sciences; Griffith University, Menzies Health Institute<br>Mnatzaganian, George; La Trobe University College of Science Health and Engineering, La Trobe Rural Health School<br>Chan, Carina ; Australian Catholic University, School of Psychology, Faculty of Health Sciences<br>Somerset, Shawn; Australian Catholic University, School of Allied Health (Department of Public Health), Faculty of Health Sciences
**Primary Subject Heading**:	Evidence based practice
Secondary Subject Heading:	Health services research, Health policy, Qualitative research
Keywords:	Barriers, knowledge translation, stroke services, evidence-based practice, Ghana, implementation science

SCHOLARONE™ Manuscripts

Barriers to evidence-based acute stroke care in Ghana: A qualitative study on the perspectives of stroke care professionals

Leonard Baatiema[1, 2*], Ama de-Graft Aikins[2], Adem Sav[3, 6], George Mnatzaganian[4], Carina K.Y. Chan[5], Shawn Somerset[3]

1. School of Allied Health, Faculty of Health Sciences, Australian Catholic University, Sydney, Australia
2. Regional Institute for Population Studies, University of Ghana, Legon, Accra, Ghana
3. School of Allied Health, Faculty of Health Sciences, Australian Catholic University, Brisbane, Australia
4. College of Science, Health and Engineering, La Trobe Rural Health School, La Trobe University, Melbourne, Australia
5. School of Psychology, Faculty of Health Sciences, Australian Catholic University, Brisbane, Australia
6. Menzies Health Institute, Griffith University, Queensland, Australia

*** Corresponding author**

Leonard Baatiema

Regional Institute for Population Studies,
University of Ghana,
P.O Box LG96
Legon-Accra, Ghana
Email Address: baatiemaleonard@gmail.com

For peer review only - http://bmjopen.bmj.com/site/about/guidelines.xhtml

Abstract

Objective

Despite major advances in research on acute stroke care interventions, relatively few stroke patients benefit from evidence-based care due to multiple barriers. Yet current evidence of such barriers is predominantly from high-income countries. This study seeks to understand stroke care professionals' views on the barriers which hinder the provision of optimal acute stroke care in Ghanaian hospital settings.

Design

A qualitative approach using semi-structured interviews. Both thematic and grounded theory approaches were used to analyse and interpret the data through a synthesis of pre-identified and emergent themes.

Setting

A multisite study, conducted in six major referral acute hospital settings (three teaching and three non-teaching regional hospitals) in Ghana.

Participants

A total of 40 participants comprising neurologists, emergency physician specialists, non-specialist medical doctors, nurses, physiotherapists, clinical psychologists and a dietitian.

Results

Four key barriers and twelve sub-themes of barriers were identified. These include barriers at the patient (financial constraints, delays, socio-cultural or religious practices, discharge against medical advice, denial of stroke), health system (inadequate medical facilities, lack of stroke care protocol, limited staff numbers, inadequate staff development opportunities), health professionals (poor collaboration, limited knowledge of stroke care interventions) and broader national health policy (lack of political will) levels. Perceived barriers varied across health professional disciplines and hospitals.

Conclusion

Barriers from low-middle income countries differ substantially from those in high income countries. For evidence-based acute stroke care in low-middle income countries such as Ghana, health policy makers and hospital managers need to consider the contrasts and uniqueness in these barriers in designing quality improvement interventions to optimise patient outcomes.

For peer review only - http://bmjopen.bmj.com/site/about/guidelines.xhtml

Keywords: Stroke care, barriers, evidence-based practice, implementation, stroke service, developing countries, Africa

Strengths and limitations of this study

- This study represents the first in Ghana to explore, in-depth, the barriers perceived by stroke care professionals to optimum provision of acute care in hospital settings.
- The work focused exclusively on the perspectives of acute stroke care professionals from diverse professional disciplines, expertise, gender, tertiary and non-tertiary hospitals across different geographic settings.
- This study did not focus on barriers to a specific stroke care intervention, as reported extensively in previous works, but rather on barriers across the continuum of stroke care.
- The study reported results from a limited set of participants whose views may not be reflective of the wider health staff responsible for acute stroke care in Ghana
- Given the qualitative nature of the study, data interpretation could be subjective and thus, caution should be applied in interpretation.

For peer review only

For peer review only - http://bmjopen.bmj.com/site/about/guidelines.xhtml

Background

Recent significant technological advancement in medical practice has increased demands, expectations and pressures on healthcare staff to provide quality and evidence-based care. This is exacerbated by the wide knowledge-clinical practice gap across the world(1),particularly in low-middle income countries(2)where research translation has become an urgent healthcare agenda(3). Empirical evidence in the USA and Europe for example, demonstrates how only about 30% to 50% of patients receive evidence-based interventions in clinical settings (4, 5). It is further suggested that translation of an evidenced-based health intervention into routine clinical practice can take up to 17 years (6).The need to identify barriers that underpin the slow uptake of evidence-based care in clinical settings is essential in understanding the extent to which health professionals provide such care to patients (5, 7). As a result, theoretical and conceptual attempts have been made to shed light on the factors which affect the current knowledge-practice gap in healthcare settings(5, 8, 9).

Due to the increasing global stroke-related mortality and morbidity (10), the past decades have witnessed a proliferation of evidence-based acute stroke care interventions (11-16).Throughout this paper, the term evidence-based acute stroke care interventions also referred to as optimal acute stroke care comprised all acute stroke care interventions based on scientific evidence, clinical judgement and expertise of a clinician and the needs of patients (17). Other key stroke experts have also recommended essential components of an evidence-based acute stroke care for improved patient outcomes (18, 19). Notwithstanding such advances, uptake of such recommendations in clinical settings remains slow(20, 21), suggesting that only a small proportion of stroke patients receive optimal care. Although the low uptake of these interventions is a global health challenge, evidence suggests the pace of uptake in high income countries exceed that of low-middle income countries (20-22). Numerous barriers have been identified to explain the low uptake of such evidence-based stroke care interventions into routine clinical practice. Some of these barriers include inadequate medical facilities, inadequate knowledge and skill levels of stroke care providers, low awareness of current acute stroke care interventions and the perceived efficacy levels of acute stroke care interventions (23-

For peer review only - http://bmjopen.bmj.com/site/about/guidelines.xhtml

25). There are also barriers at the patient level which include delays in seeking emergency care due to lack of awareness of early stroke symptoms or financial constraints (26, 27).

Although research has increased our knowledge about the range of barriers to the uptake of evidence-based stroke care in clinical settings, a more balanced and holistic understanding of such research is needed. Existing research to date only presents a one-sided view, and bias towards high income countries(e.g., Australia and USA) and moreover, is focused primarily on barriers inhibiting uptake of thrombolytic therapy (23, 25, 27, 28). However, only few studies have looked at barriers to other components of acute stroke care interventions (24, 29, 30). A study by Langhorne and colleagues also provide insightful information on the uptake of stroke unit care components in resource poor settings (20). It is unclear if these barriers apply to low-middle income countries such as Ghana where the geo-political, socio-economic and health system contexts vary. An investigation of such barriers is important in low-middle income countries because the global stroke burden is much higher there (10, 31), and yet evidence suggest uptake of evidence-based acute stroke care interventions is relatively lower (20, 21).

This study therefore aimed to identify the views of stroke care professionals on barriers inhibiting the provision of optimal acute stroke care in Ghanaian hospitals, since such information is non-existent. Acute stroke care in this context applies to the provision of care in the initial days and weeks after a stroke. Greater insights about these barriers and how they differ according to hospital settings and across stroke care professional disciplines are important for developing interventions towards enhancing optimal patient outcomes in Ghana. The findings may also have broader relevance to other resource poor settings.

Methods

Study Design

This study is part of a larger multi-site study to evaluate the provision of evidence-based acute stroke care in acute care in major referral hospitals in Ghana. A qualitative study design using semi-structured interviews was employed to gain a rich and in-depth understanding of the barriers faced by

For peer review only - http://bmjopen.bmj.com/site/about/guidelines.xhtml

stroke care professionals. The importance of qualitative data to successful translation of best scientific evidence into clinical practice has also been recommended (32). The study design, data collection, analysis and reporting were conducted in accordance with the consolidated criteria for reporting qualitative research (COREQ) (33) as shown in **Supplementary File 1.**

Settings

The study was conducted in a convenient sample of three referral tertiary (teaching) and three regional (non-teaching) hospitals from the southern, middle, and northern belts of Ghana, between November 2015 and April 2016. This represents three of the five tertiary-teaching hospitals and three of the nine regional hospitals in Ghana. The study hospitals are major referral hospitals for other hospitals and health centres located in six of the ten administrative regions of Ghana and were chosen to account for the geographic and socio-economic contrasts among the ten administrative regions of the country. The hospital bed capacity for these hospitals is as low as 150 for the regional hospital, whereas the teaching hospitals bed capacity is approximately 653. The tertiary hospitals are larger referral centres and are well-resourced with diagnostic and therapeutic facilities, whilst the regional hospitals act as major referral points to other hospitals and health centres within their catchment areas. Overall, the annual stroke admissions for 2014 ranged from 49 for the regional and 1500 stroke cases for the teaching hospitals. See **Supplementary File 3** for additional information on the study hospitals.

Research Team

LB is a Public Health PhD candidate whilst AdGA is a professor of social psychology. LB is Participants have no prior relationship with the researchers but because of the previous works of two of the researchers (LB and AdGA) in some of the study regions, it is possible participants have met or are aware of their works. LB is a health services researcher with interest in health services and policy research, research on implementation science and quality improvement interventions for stroke care health professionals. He is skilled in both qualitative and quantitative research works. AdGA conducts social and health psychology research using largely qualitative methods. AS is a health services researcher employing a mixed methods approach. The remaining researchers (GM, CKYC and SS) on the other hand, also have relevant skills, knowledge and interest in qualitative studies and the topic

For peer review only - http://bmjopen.bmj.com/site/about/guidelines.xhtml

under study. Also, AS, GM, CKYC and SS are not familiar with the study contexts and do not have any prior knowledge or relationship with participants. Overall, the research team comprised of two females and four males.

Participants

Participants comprised key hospital staff primarily involved in directing or providing acute care for stroke patients. To achieve maximum variation in the continuum of care that would reflect a real-life setting, the study recruited nurses, specialist medical doctors (neurologists, emergency physician specialist), non-specialist medical doctors, clinical psychologists, physiotherapists and a dietitian; representing diverse expertise and experience relevant to acute stroke care. **Table 1** shows participants' distribution across study sites.

Sampling and Recruitment

Purposive sampling was used to recruit all study participants. Participant recruitment was facilitated by two of the researchers (LB, AdGA), who were familiar with the study context and had significant qualitative research experience. To commence recruitment and promote the study to eligible participants, meetings were held with hospital administrators, in-service training and research coordinators, department heads and nurses in-charge in the study hospitals. Potential participants were then recommended from these meetings and engagements. Initial contact with prospective participants was made face-to-face or by telephone calls by the first author to identify the date, time and venue for the interviews. Potential participants were identified. Due to time and workload restrictions, three participants declined to participate in the study. The number of participants enrolled into the study was determined by data saturation.

Data Collection

All interviews were conducted face-to-face in English by LB who had no prior relationship with the interviewees. Data collection was conducted in various venues including: general and emergency wards, consulting rooms, conference rooms, participants' office rooms and physiotherapy departments. The interviews were facilitated by an interview guide (**Supplementary File 2**)

For peer review only - http://bmjopen.bmj.com/site/about/guidelines.xhtml

developed by the researchers and informed by an extensive literature review on the topic. The interview guide was pilot-tested with three nurses and three medical doctors at non-study sites and adapted to reflect the professional role of the interviewees. With the permission of interviewees, each interview was recorded using a digital voice recorder. Detailed field notes were also taken. The study repeatedly used prompts to facilitate the elicitation of more and clearer information or clarification of certain concepts used by participants. The interviews lasted 45 minutes on average and all recorded interviews were transcribed verbatim by professional transcribers for the final data analysis. About a third (15) of the transcripts were shared with selected participants to cross-check and ensure the information reflected the interview process and 13 transcripts were returned.

Data Analysis

Thematic data analysis (34, 35), combined with some elements of a grounded theory approach (36), were used to analyse the data. Pre-existing thematic categories based on relevant literature were utilised in the data analysis. The grounded theory, took an inductive approach to ensure all essential emergent themes from the codes not included in the deductive pre-existing coded list of barriers were captured. An initial codebook based on prior codes was developed and subsequently modified with the addition of new emergent themes after a line-by-line reading and re-reading of transcripts by one author (LB). A second author (AdGA), cross-checked the final coded results with a sample of the transcripts. Using the constant comparison approach (36, 37), a comparative analysis of both emergent and prior themes was conducted between study sites and participants to understand areas of convergence and divergence. NVivo software package version 10.0 (38) was employed to organise, code and identify all data.

Trustworthiness and transferability in the study results were facilitated by the consistent use of the interview guide during the interview process, audio recording of all interviews, professional transcription of the interviews and the use of the NVivo software to manage the entire data analysis process. As a measure to further enhance data trustworthiness(39), some transcripts were shared with a selected participants for cross checking, known as member validation..

For peer review only

Findings

A total of 40 participants took part in the study, approximately six participants per study site. Participants included both males and females of varied professional disciplines, ranks and years of practice in the study sites (**See Table 1**).

Barriers to Acute Stroke Care

Four key themes of barriers to the provision of optimal acute stroke care emerged from the data; patient, hospital or health system, health care providers' and national health policy factors. **Table 2** describes each of these barriers. Embedded in these themes were twelve sub-themes which provided specific and contextualised meaning to the main themes.

Patient factors

Under this category of barriers, five sub-themes were identified: financial constraints, delays, socio-cultural or religious beliefs and practices, discharge against medical advice and denial of stroke.

Financial constraints

In all the study sites and across participants from the various professional disciplines, barriers such as lack of funds to transport patients to the hospital, inability to pay for medical expenses (e.g., CT brain scanning services, laboratory tests and other healthcare associated expenses) were consistently raised. Patients' or their care givers' decision to first seek medical care, organise means of transport to the hospital or pay for medical expenses were often constrained by their level of financial capacity. As a result, access to care was often delayed or deprived. An excerpt from a participant emphasised this:

"poverty and ability to pay for medical cost is the issue over here,…let's say a doctor will request a patient to do a CT scan, do some lab tests …, but the patient just simply cannot afford it, or it takes too long for them to gather the money, so for two, three and sometimes five weeks you are treating a patient without a CT scan investigation" (Medical Doctor, ID 9)

Delays

Patients' late arrival to the hospital was commonly cited as another barrier to acute stroke care. Participants suggested the reasons for such delays arose from their lack of awareness of early stroke

For peer review only

symptoms and decision to first seek herbal or faith-based care, rather than medical care. Delays were also attributed to financial capacity of the family to seek medical care, especially in instances where the family breadwinner was the stroke victim. Hence, patients with good financial circumstances were more likely to seek early acute medical care compared to those with poor finances.

"…they don't bring the patients early and when they come, they will tell you the condition just started, that they just noticed the symptoms and rushed the patient to the hospital. But you realize that this patient had the stroke for long, not very acute as they described, either they have sought treatments elsewhere or other interventions before arriving here" (Nurse, ID 4)

On the low awareness of early stroke symptoms, one participant noted:

"They don't have knowledge of early stroke symptoms, they are ignorant about stroke symptoms…, Because they don't know what the condition is, patients or families will rather prefer to self-medicate with painkiller or remain at home upon symptoms onset with the hope that the symptoms will disappear" (Medical Doctor, ID2)

Socio-cultural and religious beliefs

Patients' socio-cultural or religious beliefs and practices emerged as another predominant barrier. Cultural beliefs and practices (e.g. view stroke having a spiritual cause, retribution from their gods and not a condition which can be managed medically) were often very important and likely to influence patient health seeking behaviour. A common practice from such beliefs was patients' desperate attempts to defer medical care for herbal or traditional medical care or make attempts to combine both whilst hospitalised. Some nurses noted such practices or beliefs have often compelled families of patients to abandon medical care in the hospital for alternative care provided by traditional or faith healers. For example, all physiotherapists interviewed believed socio-cultural beliefs and practices have limited patient attendance of outpatient care after discharge as most resorted to local herbal treatment options or to prayer camps. A participant had this to say about the religious beliefs on treatment compliance:

For peer review only - http://bmjopen.bmj.com/site/about/guidelines.xhtml

".....they become very spiritual once they are diagnosed with a stroke; most now want to focus on their spiritual life instead. You realize that consistently our stroke patients want to talk about God, talking about how lucky they have been, how God has saved them from death" (Clinical Psychologist, ID 1)

Discharge against medical advice

'Discharge against medical advice' was consistently cited by participants and emerged as a key barrier to optimal acute stroke care. This practice was generally perpetuated by two factors; financial capacity to meet medical expenses and families' desire to resort to other forms of care such as traditional herbal medicine, consultation of spiritualists or faith healers. Participants attributed the increased patient and family interest to such alternative forms of care to the vibrant advertisements across the media by traditional herbal medicine practitioners and faith healers. Indeed, promises were made by such individuals to cure stroke and other chronic conditions within a week or two after commencing treatment:

"I recently heard one advertisement which said acute stroke patients should just come here and will be made to walk within a week. So it has gotten to the point where patients easily get misled by these adverts, they find these traditional or faith healers attractive and accessible" (Medical Doctor, ID 11)

According to nurses, such incidences were also linked to the socio-cultural beliefs and practices of the people where health conditions such as stroke, were associated with supernatural or spiritual causes. After being informed about their stroke condition, some stroke patients often insisted to be discharged. Moreover, refusal to heed patients or families' requests for early discharge often resulted in non-compliance to treatment, sudden disappearance of patients or desertion of patients by family members:

"with the relatives, as soon as they find out that it is a stroke, they start finding ways of transferring the patient to seek herbal medication or to a prayer camp ..., so they request for discharge against medical advice and take the patient away" (Nurse, ID 15)

Denial

For peer review only - http://bmjopen.bmj.com/site/about/guidelines.xhtml

It was also reported that some family members or patients sometimes rejected the diagnosis and dissociated their condition from stroke after being informed about the condition. Participants even acknowledged instances where some family members challenged their professional competence because they felt an incorrect diagnosis was made. The denial of stroke stemmed from the diverse misunderstandings of the illness, with some patients/family members viewing it as an attack or retribution from their gods or spirits for a wrong doing. In such situations, the provision of care was difficult, as some family members were less compliant during treatment:

"I remember one care giver following up to me to inquire whether we were sure the condition of their relative was a stroke, as she believed a wrong diagnosis was documented. Because to her, their relative does not deserve to have a stroke" (Nurse, ID 11)

Hospital or Health system factors

The subthemes of these system factors were shortage of medical facilities/equipment, lack of a stroke specific protocol, inadequate staff numbers and limited staff professional development opportunities.

Shortage of medical facilities

The limited availability of essential medical equipment to facilitate effective provision of acute stroke care was a common feature in study hospitals within the northern belt. There was a shortage of medical facilities such as blood pressure (BP) monitoring apparatus, cardio monitors, suction machines, adjustable hospital beds and inadequate space to facilitate patient care. For example, participants in the only stroke unit in this study believed that the inadequate bed capacity (6-bed capacity) limited admission of many patients to receive optimal care. One participant commented:

"Unfortunately, you find stroke patients, they come in, no bed, they are sitting on chairs, sitting on the wheel chair or on the bare floors, these are the conditions under which we are expected to provide standard care...."(Medical Doctor, ID 2)

This experience and another comment below exemplified this barrier:

For peer review only

"We have just one oxygen for all the patients in this ward so the nurses are sometimes compelled to use their discretion to wean patients off oxygen to enable another patient benefit if his/her condition is more severe" (Medical Doctor, ID 7)

Additionally, the lack of a stroke unit was a common concern expressed by medical doctors from hospitals in the middle and southern belt, a situation they believed was caused by limited funds allocated by hospitals and a low priority for acute stroke care. A lack of medical equipment and consumables could delay or deprive patients of standard care. Participants also cited instances where some medical doctors acquired personal blood pressure monitoring devices to support patient care because of shortages. Another issue was the absence or frequent malfunction or breakdown of diagnostic services such as CT scanning services, a situation which often delayed care delivery or led to referral of patients to other hospitals. According to some medical doctors, this situation sometimes compelled them to proceed with care delivery without a CT scan investigation to inform treatment options:

"I can say the biggest problem we face is our diagnostic equipment. See the whole of this so called big hospital, we have only one CT scan machine. The machine has been out of service for over 6 to 8 weeks and was only put to use again two weeks ago …," (Medical Doctor, ID 11)

Lack of a specific protocol for acute stroke care

Most nurses believed the absence of a specific protocol or clinical guideline for acute stroke care was a key barrier.

"….sometimes the cases come and you've forgotten some important procedures because I left the classroom a very long time ago" (Nurse, ID 13)

One nurse recounted her experience of providing an acute stroke care with much uncertainty because there was no medical doctor or a senior colleague to guide her. This nurse stressed the importance of a clinical protocol, which she argued could facilitate the provision of standardised care even in the absence of a specialist or a medical doctor:

For peer review only - http://bmjopen.bmj.com/site/about/guidelines.xhtml

"Most of the stroke cases I have witnessed were rushed in here during late hours, sometimes after mid-night and most times, its only nurses present to attend to the case. So the patient has to wait until a doctor arrives, sometimes the next morning and that is why I think the protocol will at least guide us to safely initiate initial treatment'' (Nurse, ID 18)

Limited staff

Limited staff especially stroke specialists (e.g. neurologists, neurosurgeons and trained stroke nurses) were also a key barrier across the study sites. This issue was more dominant in the non-tertiary regional hospitals and participants in the northern belt of Ghana. Participants, especially nurses, believed the current staff numbers were inadequate to provide optimal acute stroke care (e.g. regular checking of BP levels, sugar levels, regular turning of patients to prevent pressure sores and management of urinal incontinence to minimise risk of urinary tract infections).They expressed frustration about the high work load, which often compromised effective patient care:

"*you could have patients running over 40,…, some are in the wheel chair, some are on the chairs you see over there, some are on the beds, sometimes some are on the stretchers'' (Nurse, ID 5)*

Limited staff professional development opportunities

With the exception of medical doctors, nurses and allied health staff expressed great interest in opportunities for staff professional development; mainly in hands-on training workshops related to stroke clinical care. Although there were policies to support staff develop their current knowledge and skills, such opportunities were very rare. Nurses, for example, emphasised the importance of continuous education and professional development as current clinical practice was underpinned by what they were taught in schools many years ago. Overall, there was strong opinion on this matter and a lack of continuous training opportunities inherently affected the quality of care provided to acute stroke patients:

"we don't have regular workshops…, even if there will ever be such an opportunity, you will only consider attending provided you can afford the cost as this hospital won't support us attend such a workshop" (Nurse, ID 4)

For peer review only - http://bmjopen.bmj.com/site/about/guidelines.xhtml

Health care providers' factors

Two main subthemes of barriers were identified at the health care staff level; limited knowledge in acute stroke care and inadequate team collaboration and coordination.

Inadequate knowledge

Lack of knowledge on how to provide appropriate treatment was often discussed, particularly by nurses. Unlike the medical doctors, all the nurses interviewed were unware of thrombolytic therapy. This particular type of therapy was not part of what the medical doctors recommended for acute ischemic stroke care:

"What did you say again? thromboly… what? Not here, I am hearing thrombolysis for the first time. It is not part of our treatment plan for stroke patients in this hospital. How come you say it is one of the key therapies for acute ischemic stroke and I am not aware of it?'' (Nurse, ID 9)

Most nurses also identified insufficient knowledge of certain acute stroke care procedures as a barrier, especially in triaging unconscious stroke patients. They expressed uncertainty about their ability to often proceed with care delivery in the absence of a medical doctor. Although nurses talked about consulting senior colleagues, some level of uncertainty was still noted in proceeding to provide care in the absence of a medical doctor. Despite their clinical training, nurses cited difficulties arising from efforts to respond to unconscious stroke patients and conduct assessment to support an accurate stroke prognosis:

"Sometimes a stroke case arrives unconscious and you start shivering especially when it is in the night and there is no doctor around to respond immediately. I feel very nervous when I realise I am the only senior nurse in the ward to attend to this patient" (Nurse, ID 12)

Team Collaboration and Communication

According to most nurses and all allied health staff, collaborative work in a multidisciplinary stroke team was inadequate, and an obstacle to effective patient care. Physician driven stroke care without adequate involvement of other staff, was frequently discussed.

For peer review only - http://bmjopen.bmj.com/site/about/guidelines.xhtml

"I don't even think we have a working team here, it is more of a doctor giving instructions…,giving instructions to nurses, though nurses are there with the patients 24 hours, the medical doctors just come to see their patients and then disappear. Is that what you call teamwork?" (Nurse, ID 14)

Allied health staff expressed a sense of marginalisation and disconnectedness, especially in the early stages of care. A dietitian for example cited instances where medical teams (doctors and nurses) often discharge patients without his view on dietary plans at discharge. Three physiotherapists expressed similar concerns of limited involvement which in their view inhibited their ability to develop initial rapport with patients or the opportunity to educate patients about the importance of self-care practices, following discharge:

"There was an occasion my rounds coincided with the medical team's rounds; I quickly joined them. I made a suggestion on a particular patient we were attending to but this was brushed off and the medical doctor behaved as if I was trying to direct him what to do or take over his job" (Physiotherapist, ID 3)

National policy context factors

Participants identified one key barrier under this theme; lack of political will for acute stroke care.

Lack of Political Will

The lack of national level support and political will for acute stroke care was consistently cited as a broad level barrier, particularly by medical doctors. They expressed strong views on this issue, attributing it to the increasing out-of-pocket medical expenses for patients. Despite the existence of the national health insurance policy which was supposed to replace the practice of 'cash and carry', a lack of political will for the scheme has gradually introduced the policy of upfront payments by patients prior to acute care in most hospitals in Ghana presently. They believed this has negatively affected patients' access to care (not only stroke patients) because of their inability to pay for medical expenses. The limited coverage of the national health insurance scheme on chronic care, such as stroke, was also stated as a key barrier. Patients' experienced difficulties paying for stroke-related medical costs (e.g., CT brain scans and other laboratory tests) that were not covered by the national

For peer review only - http://bmjopen.bmj.com/site/about/guidelines.xhtml

health insurance scheme. Overall, there was a sense of powerlessness about national level neglect for acute stroke care. Consequently, this resulted in staff dissatisfaction and a lack of motivation to provide effective care.

"The problems we face in our current health sector has very little to do with health professionals' reluctance to provide standard care. It is the health system!..., we are under a system where every medication is expensive for the ordinary Ghanaian to afford and yet, we make the patients to believe the national health insurance policy covers everything. Now almost every medication has to be paid for by the patient and if they can't afford what we recommend as best treatment option for their condition, we provide the alternatives which may not be very effective" (Medical Doctor, ID 11).

Discussion

Summary of main findings

This study provides in-depth insights of the barriers to the delivery of optimal acute stroke care in Ghana, a largely neglected low-middle income country in Africa. The findings suggest that although the barriers identified share some commonality with those reported in previous studies in high income countries, some barriers are unique to optimal stroke care in low-middle income settings such as Ghana. Some of the predominant barriers to acute stroke care in high income countries often comprised patient delay in seeking early care, inadequate medical facilities to support optimal patient care, healthcare providers' attitudes towards some acute stroke care interventions, poor communication and lack of cooperation among healthcare providers (23-25). On the contrary, although there is an overlap of these barriers in both high income and low-middle income countries, the issue of discharge against medical advice and the role of socio-cultural and religious beliefs or practices of stroke patients and their families characterise the present study. Whilst illuminating the reasons influencing the provision of optimal acute stroke care in a typical resource poor setting, the findings further unravel barriers peculiar to different stroke care professionals and hospital settings where much policy attention is required for effective and timely translation of evidence-based stroke care intervention into routine clinical practice.

For peer review only - http://bmjopen.bmj.com/site/about/guidelines.xhtml

Comparison with previous literature

As found in this study, stroke care in high income countries with modern resources has consistently reported barriers corresponding to patient, health system/hospital, health staff and the national level factors (23, 25, 40). Highlighting patient level barriers as the most predominant of all barriers identified in our study, a previous study corroborated this by reporting91% of participants viewed prehospital delay at the patient level as the most dominant barrier to providing thrombolytic therapy (27). An earlier study in Ghana (41) found that only 40% (277/693) could correctly identify stroke symptoms, also reinforces the importance of our finding that participants identified patient delays to seek care due to low awareness of stroke symptoms.

Although our findings on the importance of patient-level barriers to optimal stroke care, are in line with previous research (23, 27, 30, 42),the explanation and the circumstances in which some patient level factors acted as barriers to optimal acute stroke care were somewhat different in this study. For example, socio-cultural or religious beliefs and practices were perceived to underpin health seeking behaviours of stroke patients and their families in Ghana. Although this is inconsistent with the literature on barriers to acute stroke care in high income countries, our findings corroborate with research on other chronic diseases and health seeking behaviour in Ghana (43, 44).This underscores the influence such beliefs and practices to health seeking behaviours of patients and families in Ghana. Evidence within the African contexts suggest patient access to traditional and faith healers as complimentary avenues of care is due to the easy access, lower cost and cultural legitimacy of such alternatives (45, 46).

In addition, patient discharge against medical advice was also a key barrier affecting optimal clinical care. This finding is also largely inconsistent with published barriers to acute stroke care from high income countries. Despite the limited popularity of such barriers in previous studies, this has been well articulated in other health contexts and conditions with conclusive arguments of the practice being a drawback and an obstacle to provision of adequate and quality health care (47-50). Clearly, this issue requires further investigation in Ghana, and possibly other low-middle income countries. Larger studies would clarify the extent to which this can affect standard acute stroke care.

For peer review only - http://bmjopen.bmj.com/site/about/guidelines.xhtml

Other important barriers from this study were related to the health system such as limited stroke care specialists, increased workload for staff, inadequate medical facilities, lack of protocols and unavailability or limited access to CT brain scans. The importance of this set of barriers has been reported previously (23, 29, 51), highlighting the extent to which they affect provision of optimal patient care. For example, one study found that 71% of participants identified lack of protocols, care paths and opportunities for staff professional education as important barriers to the provision of optimal acute stroke care (27). Comparable to our study, a Swedish study identified low staffing levels as a major barrier to optimal stroke care (25). Despite these studies being conducted in high income countries, their corroboration with the present study reinforces the importance of hospital/health system level barriers to the uptake of evidence-based practice.

The issue of limited collaboration or involvement of allied health staff and other providers in the provision of care is also worthy of attention. Multidisciplinary and coordinated care remains a central component in contemporary evidence-based practice for acute stroke care (18, 52). As a result, inadequate involvement of these staff is a significant issue since participants noted that their limited involvement is detrimental to optimal patient care. Evidence from existing scholarship on such barriers has been previously reported (53-55), thus stressing the need to consider interventions to improve collaboration among stroke care professionals in acute stroke care.

Nurses' knowledge of acute stroke care interventions such as thrombolytic therapy was also identified as a barrier, consistent with previous studies (23, 27, 40). This issue has also been identified in an Australian study where 50% of nurses reported having limited knowledge of thrombolytic therapy (27). Such findings highlight the importance of this issue to optimal stroke care in both low-middle-income and high-income countries.

Finally, another barrier identified in the present study relates to the low level of political will for optimal acute stroke care. This barrier is evident in the absence of a national stroke clinical guideline, a national framework for quality improvement interventions for stroke and limited coverage of the national health insurance scheme to cover patients' medical expenses. Whilst this finding corroborates with previous studies asserting the limited prioritization of acute stroke care by health policy makers

For peer review only - http://bmjopen.bmj.com/site/about/guidelines.xhtml

in resource poor settings (56-59), this could also likely be symptomatic of the current limited global health funding for stroke and other non-communicable diseases compared to communicable diseases (60).

Implications for future research, policy and clinical practice

The present findings have several important implications for the provision of evidence-based acute stroke care in Ghana. Firstly, patient financial constraints appear to be a key barrier to optimal care and needs urgent attention. It is apparent that patients and family members struggle with the financial costs of stroke treatment and strategies are needed to overcome this burden. The current Ghana National Health Insurance Policy offers limited financial risk protection for stroke care. This epitomises the fragile nature of health care systems in low-middle income countries, which require significant structural and policy reforms to minimise the current high cost of treatment for chronic diseases such as stroke. If left unaddressed, the consequent increased incidence of late arrival or refusal to seek care, limited treatment options and patient discontinuation of treatment will negatively affect optimal patient outcome.

Secondly, the evidence of socio-cultural or religious beliefs and practices as a barrier to optimal care also deserves attention, particularly since this has received little attention in the current literature on barriers to acute stroke care. More research in other settings may be useful to unravel the extent to which such practices influence provision of optimal care. In addition, national and local public awareness campaigns to increase the health literacy levels of the populace regarding stroke risk factors, early stroke symptoms and the need to seek early medical care are critical. This level of public awareness and education campaigns have been implemented in high income countries such as UK (61, 62), Australia (63, 64), Canada (65) and Germany (66). Hence Ghana and other low-middle-income countries can clearly draw lessons from such public awareness campaigns on early recognition of stroke symptoms to minimise patient delays to seek care. Such interventions however would need to be adapted to suit the particular country and health context. They should be mainstreamed in the healthcare systems of such countries through collaboration with the public and private sectors to optimise the impact.

For peer review only - http://bmjopen.bmj.com/site/about/guidelines.xhtml

Importantly, the finding of patient discharge against medical advice has revived debates about the place of patients within the current evidence-based medicine paradigm where patient's needs and preferences are essential (67). Whilst more research on the implications of this issue is required, institutional measures exploring safe and appropriate times and conditions under which such requests could be granted should be identified. More importantly, strategies need to be adopted to ensure that requests by patients and families to be discharged against medical advice be counterbalanced with tailored communication and public campaigns to improve awareness of the risks and benefits. The roles of clinical psychologists and nurses can be pivotal in such communication. This has the potential to minimise the incidence of patient discharge against medical advice.

The limited collaboration and poor communication among stroke care professionals also warrants attention. Highlighted as an imperative in providing optimal acute stroke care by previous research (12, 54, 55), the finding in this study further emphasises the need to explore effective ways to build collaborative working environments. Structural policy reforms are needed to ensure equal respect for individual professional experiences, identity, autonomy and responsibilities. This may be in the form of healthcare professional trainings, educational meetings and conferences, workshops to explore ways of improving clinical outcomes. As indicated earlier, staff professional development plays a critical role to stroke care quality improvement (68) and overall health outcomes(69), and as such, efforts to provide staff educational and professional development opportunities in stroke care could be useful in the Ghanaian setting.

Given that health system and hospital level factors were observed as important to stroke care, strengthening health systems through the provision of adequate and effective acute stroke care services is essential. For example, to address the issue of limited staff numbers, an immediate short term measure by health managers and policy makers would be to consider task shifting approaches, as has been trialled in Nigeria. This Nigerian study (70) showed improved knowledge of non-neurologists in acute stroke care, thus potentially translating into improved patient outcomes.

Another health system barrier which has critical implications is the reported arrangement of upfront payment by patients prior to delivery of health care services such as laboratory test brain scanning

For peer review only - http://bmjopen.bmj.com/site/about/guidelines.xhtml

services. This suggest that people with symptoms of stroke and other emergency conditions such as heart attack and asthma may be provided optimal care on condition the patient is able to pay for such services. A health policy effort to expand the package of the current Ghanaian health insurance policy to be more comprehensive to cover the cost of CT brain scanning services and other medical services not covered under the insurance policy as discussed earlier. In line with this, regular reimbursement of claims by the appropriate state institutions may address the issue of upfront payment prior to care.

Finally, to increase implementation of evidence-based acute stroke care, there is the need for increased policy commitment for optimal acute stroke care through increased allocation of resources to hospitals in the form of infrastructural support, a comprehensive coverage of the current national health insurance policy to include CT brain scan services and medical expenses for chronic care, staff professional development opportunities, and development of a stroke specific clinical guideline are urgently needed.

Limitations and Strengths

As a limitation, this study reported results from a limited set of participants whose views may not be reflective of the wider health staff responsible for acute stroke care in Ghana. Nonetheless, this was conducted in six major referral hospitals in six of the ten administrative regions of Ghana and so the findings may be applicable to other stroke care professionals. Future studies should target a larger and more representative study sample to also include health planners and administrators in district and municipal hospitals. Further, given the qualitative nature of the study, the use of a semi-structured interview guide and data interpretation could be subjective and thus, caution should be applied in interpretation. Nevertheless, using a robust reporting guideline, participant cross checking and validation of interview transcripts, and the consistent use of the interview guide during the interview process minimises any possibility of bias but rather enhanced the study validity and reliability. Another key limitation is the lack of observational or documentary evidence which could have accounted for any potential important information which may not have been shared by the participants during the interview process.

For peer review only - http://bmjopen.bmj.com/site/about/guidelines.xhtml

Notwithstanding these limitations, this study also has several strengths. First, to our knowledge, this study represents the first in Ghana to explore, in-depth, the barriers perceived by stroke care professionals to optimum provision of acute care in hospital settings. Another key strength is its exclusive focus on the perspectives of acute stroke care professionals from diverse professional disciplines, expertise, gender, tertiary and non-tertiary hospitals across different geographic settings. Using qualitative design, the findings provide contextually rich information of such barriers which would have been more difficult to unravel quantitatively. The study findings provide some new insights of other factors which have been less recognised in previous literature on the barriers to evidence-based acute stroke care. Added to this, this study did not focus on barriers to a specific stroke care intervention, as reported extensively in previous works, but rather on barriers across the continuum of stroke care.

Conclusion

Overall, the views on barriers to optimal stroke care varied significantly based on specific professional discipline and study sites. Although most of the barriers were largely consistent with previous studies in high income countries, the study unravelled some unique barriers which extend the body of literature on barriers to acute stroke care. Importantly, barriers in low-middle income countries showed important differences to those from high income countries. Greater political will for acute stroke care in terms of increased coverage of the national health insurance scheme, increased resource allocation, recruitment and training of an expanded stroke health workforce could improve uptake of evidence-based acute stroke care interventions. The information provided in this paper is potentially important to health managers, policy makers, patients, grant managers or holders and other health stakeholders as it presents various reasons why delivery of acute stroke care in clinical setting may be far from optimum. To this end, to translate current evidence-based acute stroke interventions for optimal patient outcomes in Ghana and potentially in other resource poor settings, a clear-cut understanding of these barriers to inform policy formulation, quality improvement and staff professional development is critical.

For peer review only - http://bmjopen.bmj.com/site/about/guidelines.xhtml

For peer review only

Acknowledgement

The authors gratefully acknowledge the support received from the staff of the study hospitals during field data collection. The contribution of Dr Michael Otim, Associate Professor Liz Mclnnes and Professor Sandy Middleton to the study conception are greatly acknowledged.

Authors Contribution

Study design and instrumentation process: LB, AdGA, GM, SS. Participants recruitment: LB, AdGA. Data collection, analysis, interpretation and writing of first manuscript: LB. Contribution to data interpretation and critical review of the manuscript: LB, AdGA, AS, GM, CKYC and SS. All authors have made substantial contribution to the writing of this manuscript for critical intellectual content. All authors have reviewed and approved the final version for submission.

Funding

The first author (LB) is a PhD Candidate funded under the University International Students Scholarship programme. LB also received funding support from the University Faculty of Health Science Higher Degree Research (HDR) Student Support Scheme during his candidature. However, these funding bodies did not play a role in the study design, data collection and analysis, results interpretation, writing of the entire manuscript and the decision to submit the manuscript to this journal.

Competing Interest: The authors declare no competing interests

Ethics

This study received ethical approval from the University Human Research Ethics Committee (2015-154H), the Ghana Health Service Ethical Review Committee on Research Involving Human Subjects (GHS-ERC: 11/07/15) and the Committee on Human Research Publications and Ethics of the School of Medical Sciences of the Kwame Nkrumah University of Science and Technology and the Komfo Anokye Teaching Hospital (CHRPE/AP/141/16). The Institutional Review Board of the 37 Military Hospital (37MH-IRB IPN 035/2015) also granted clearance for the conduct of this study.

Data sharing statement: No additional data are available

For peer review only - http://bmjopen.bmj.com/site/about/guidelines.xhtml

Reference

1. World Health Organization, editor Bridging the "Know–Do" Gap. Meeting on knowledge translation in global health; 2006: Gene-va: WHO.
2. Hutin YJ, Hauri AM, Armstrong GL. Use of injections in healthcare settings worldwide, 2000: literature review and regional estimates. Bmj. 2003;327(7423):1075.
3. Chan CK, Oldenburg B, Viswanath K. Advancing the Science of Dissemination and Implementation in Behavioral Medicine: Evidence and Progress. International journal of behavioral medicine. 2015;22(3):277-82.
4. Schuster MA, McGlynn EA, Brook RH. How good is the quality of health care in the United States? Milbank Quarterly. 2005;83(4):843-95.
5. Grol R, Wensing M, Eccles M, Davis D. Improving patient care: the implementation of change in health care: John Wiley & Sons; 2013.
6. Morris ZS, Wooding S, Grant J. The answer is 17 years, what is the question: understanding time lags in translational research. Journal of the Royal Society of Medicine. 2011;104(12):510-20.
7. Hamilton S, McLaren S, Mulhall A. Assessing organisational readiness for change: use of diagnostic analysis prior to the implementation of a multidisciplinary assessment for acute stroke care. Implementation Science. 2007;2(1):1.
8. Straus S, Tetroe J, Graham ID. Knowledge translation in health care: moving from evidence to practice: John Wiley & Sons; 2013.
9. SURE Collaboration. SURE Guides for Preparing and Using Evidence-Based Policy Briefs: 5 Identifying and addressing barriers to implementing policy options. 2011.
10. Mensah GA, Norrving B, Feigin VL. The global burden of stroke. Neuroepidemiology. 2015;45(3):143-5.
11. Sandercock P, Counsell C, Tseng M, Cecconi E. Oral antiplatelet therapy for acute ischaemic stroke (Review). Cochrane Database Syst Rev. 2014(3).
12. Trialists'Collaboration SU. Organised inpatient (stroke unit) care for stroke. Cochrane Database Syst Rev. 2013;9.
13. Wardlaw JM, Murray V, Berge E, del Zoppo GJ. Thrombolysis for acute ischaemic stroke. The Cochrane Library. 2014.
14. Vahedi K, Hofmeijer J, Juettler E, Vicaut E, George B, Algra A, et al. Early decompressive surgery in malignant infarction of the middle cerebral artery: a pooled analysis of three randomised controlled trials. The Lancet Neurology. 2007;6(3):215-22.
15. Donnan GA, Davis SM, Parsons MW, Ma H, Dewey HM, Howells DW. How to make better use of thrombolytic therapy in acute ischemic stroke. Nature Reviews Neurology. 2011;7(7):400-9.
16. Donnan GA, Fisher M, Macleod M, Davis SM. Stroke. The Lancet. 2008;371(9624):1612-23.
17. Baatiema L, Otim M, Mnatzaganian G, Aikins AD-G, Coombes J, Somerset S. Towards best practice in acute stroke care in Ghana: a survey of hospital services. BMC Health Services Research. 2017;17(1):108.
18. Langhorne P, Pollock A. What are the components of effective stroke unit care? Age and Ageing. 2002;31(5):365-71.
19. Lindsay P, Furie KL, Davis SM, Donnan GA, Norrving B. World Stroke Organization Global Stroke Services Guidelines and Action Plan. International Journal of Stroke. 2014;9:4-13.
20. Langhorne P, de Villiers L, Pandian JD. Applicability of stroke-unit care to low-income and middle-income countries. The Lancet Neurology. 2012;11(4):341-8.

For peer review only - http://bmjopen.bmj.com/site/about/guidelines.xhtml

21. Berkowitz AL, Mittal MK, McLane HC, Shen GC, Muralidharan R, Lyons JL, et al. Worldwide reported use of IV tissue plasminogen activator for acute ischemic stroke. International Journal of Stroke. 2014;9(3):349-55.
22. Rhoda A, Smith M, Putman K, Mpofu R, DeWeerdt W, DeWit L. Motor and functional recovery after stroke: a comparison between rehabilitation settings in a developed versus a developing country. BMC health services research. 2014;14(1):82.
23. Meurer WJ, Majersik JJ, Frederiksen SM, Kade AM, Sandretto AM, Scott PA. Provider perceptions of barriers to the emergency use of tPA for acute ischemic stroke: a qualitative study. BMC emergency medicine. 2011;11(1):5.
24. Purvis T, Moss K, Denisenko S, Bladin C, Cadilhac DA. Implementation of evidence-based stroke care: enablers, barriers, and the role of facilitators. Journal of multidisciplinary healthcare. 2014;7:389.
25. Stecksén A, Lundman B, Eriksson M, Glader E-L, Asplund K. Implementing Thrombolytic Guidelines in Stroke Care Perceived Facilitators and Barriers. Qualitative health research. 2013:1049732313514137.
26. Hargis M, Shah JN, Mazabob J, Rao CV, Suarez JI, Bershad EM. Barriers to administering intravenous tissue plasminogen activator (tPA) for acute ischemic stroke in the emergency department: A cross-sectional survey of stroke centers. Clinical Neurology and Neurosurgery. 2015;135:79-84.
27. Williams JM, Jude MR, Levi CR. Recombinant tissue plasminogen activator (rt-PA) utilisation by rural clinicians in acute ischaemic stroke: A survey of barriers and enablers. Australian Journal of Rural Health. 2013;21(5):262-7.
28. Grady A, Bryant J, Carey M, Paul C, Sanson-Fisher R. Enablers of the Implementation of Tissue Plasminogen Activator in Acute Stroke Care: A Cross-Sectional Survey. PloS one. 2014;9(12):e114778.
29. O'Rourke F, Chan DKY, Chan DL, Ding XM. Preferences, barriers and facilitators for establishing comprehensive stroke units: a multidisciplinary survey. Australian Health Review. 2013;37(3):318-23.
30. Van Der Weijden T, Hooi JD, Grol R, Limburg M. A multidisciplinary guideline for the acute phase of stroke: barriers perceived by Dutch neurologists. Journal of evaluation in clinical practice. 2004;10(2):241-6.
31. Feigin VL, Lawes CM, Bennett DA, Barker-Collo SL, Parag V. Worldwide stroke incidence and early case fatality reported in 56 population-based studies: a systematic review. The Lancet Neurology. 2009;8(4):355-69.
32. Greenhalgh T, Howick J, Maskrey N. Evidence based medicine: a movement in crisis? 2014.
33. Tong A, Sainsbury P, Craig J. Consolidated criteria for reporting qualitative research (COREQ): a 32-item checklist for interviews and focus groups. International Journal for Quality in Health Care. 2007;19(6):349-57.
34. Braun V, Clarke V. Using thematic analysis in psychology. Qualitative research in psychology. 2006;3(2):77-101.
35. Bryman A. Social research methods: Oxford university press; 2012.
36. Strauss A, Corbin J. Basics of qualitative research: Techniques and procedures for developing grounded theory: Sage Publications, Inc; 1998.
37. Bradley EH, Curry LA, Devers KJ. Qualitative data analysis for health services research: developing taxonomy, themes, and theory. Health services research. 2007;42(4):1758-72.
38. QSR International. NVivo Version 10.0 [Computer software] 2015 [Available from: http://www.qsrinternational.com/.

For peer review only - http://bmjopen.bmj.com/site/about/guidelines.xhtml

39. Mays N, Pope C. Assessing quality in qualitative research. British medical journal. 2000;320(7226):50.
40. Donnellan C, Sweetman S, Shelley E. Health professionals' adherence to stroke clinical guidelines: a review of the literature. Health policy. 2013;111(3):245-63.
41. Donkor E, Owolabi M, Bampoh P, Aspelund T, Gudnason V. Community awareness of stroke in Accra, Ghana. BMC Public Health. 2014;14(1):196.
42. Slot KB, Murray V, Boysen G, Berge E. Thrombolytic treatment for stroke in the Scandinavian countries. Acta Neurologica Scandinavica. 2009;120(4):270-6.
43. Aikins Ad-G. Healer shopping in Africa: new evidence from rural-urban qualitative study of Ghanaian diabetes experiences. Bmj. 2005;331(7519):737.
44. Teye A. Psychosocial Well-Being of Individuals Living with Chronic Non-Communicable Diseases in Urban Poor Communities of Ghana: University of Ghana; 2013.
45. Awah PK, Unwin N, Phillimore P. Cure or control: complying with biomedical regime of diabetes in Cameroon. BMC Health Services Research. 2008;8(1):1.
46. Marshall IJ, Wolfe CD, McKevitt C. Lay perspectives on hypertension and drug adherence: systematic review of qualitative research. 2012.
47. Choi M, Kim H, Qian H, Palepu A. Readmission rates of patients discharged against medical advice: a matched cohort study. PloS one. 2011;6(9):e24459.
48. Glasgow JM, Vaughn-Sarrazin M, Kaboli PJ. Leaving against medical advice (AMA): risk of 30-day mortality and hospital readmission. Journal of general internal medicine. 2010;25(9):926-9.
49. Southern WN, Nahvi S, Arnsten JH. Increased risk of mortality and readmission among patients discharged against medical advice. The American journal of medicine. 2012;125(6):594-602.
50. Garland A, Ramsey CD, Fransoo R, Olafson K, Chateau D, Yogendran M, et al. Rates of readmission and death associated with leaving hospital against medical advice: a population-based study. Canadian Medical Association Journal. 2013;185(14):1207-14.
51. Hamilton S, McLaren S, Mulhall A. Assessing organisational readiness for change: use of diagnostic analysis prior to the implementation of a multidisciplinary assessment for acute stroke care. Implementation Science. 2007;2.
52. Trialists'Collaboration SU. Organised inpatient (stroke unit) care for stroke. Cochrane database of systematic reviews. 2013(4).
53. Bayley MT, Hurdowar A, Richards CL, Korner-Bitensky N, Wood-Dauphinee S, Eng JJ, et al. Barriers to implementation of stroke rehabilitation evidence: findings from a multi-site pilot project. Disability and rehabilitation. 2012;34(19):1633-8.
54. Gibbon B, Watkins C, Barer D, Waters K, Davies S, Lightbody L, et al. Can staff attitudes to team working in stroke care be improved? Journal of advanced nursing. 2002;40(1):105-11.
55. Baxter SK, Brumfitt SM. Professional differences in interprofessional working. Journal of Interprofessional Care. 2008;22(3):239-51.
56. Cadilhac D, Lalor E, Pearce D, Levi C, Donnan G. Access to stroke care units in Australian public hospitals: facts and temporal progress. Internal Medicine Journal. 2006;36(11):700-4.
57. Fuster V, Voute J, Hunn M, Smith SC. Low Priority of Cardiovascular and Chronic Diseases on the Global Health Agenda A Cause for Concern. Circulation. 2007;116(17):1966-70.
58. Rudd A, Hoffman A, Down C, Pearson M, Lowe D. Access to stroke care in England, Wales and Northern Ireland: the effect of age, gender and weekend admission. Age and Ageing. 2007;36(3):247-55.
59. Aikins Ad-G, Boynton P, Atanga LL. Review Developing effective chronic disease interventions in Africa: insights from Ghana and Cameroon. 2010.

For peer review only - http://bmjopen.bmj.com/site/about/guidelines.xhtml

60. Institute for Health Metrics and Evaluation. Financing Global Health 2015 :Development assistance steady on the path to new Global Goals. Seattle, WA: Institute for Health Metrics and Evaluation; 2016.

61. Dombrowski SU, Mackintosh JE, Sniehotta FF, Araujo-Soares V, Rodgers H, Thomson RG, et al. The impact of the UK 'Act FAST'stroke awareness campaign: content analysis of patients, witness and primary care clinicians' perceptions. BMC public health. 2013;13(1):915.

62. Flynn D, Ford GA, Rodgers H, Price C, Steen N, Thomson RG. A time series evaluation of the FAST national stroke awareness campaign in England. PloS one. 2014;9(8):e104289.

63. Bray JE, Mosley I, Bailey M, Barger B, Bladin C. Stroke public awareness campaigns have increased ambulance dispatches for stroke in Melbourne, Australia. Stroke. 2011;42(8):2154-7.

64. Bray JE, Johnson R, Trobbiani K, Mosley I, Lalor E, Cadilhac D. Australian public's awareness of stroke warning signs improves after national multimedia campaigns. Stroke. 2013;44(12):3540-3.

65. Hodgson C, Lindsay P, Rubini F. Can mass media influence emergency department visits for stroke? Stroke. 2007;38(7):2115-22.

66. Worthmann H, Schwartz A, Heidenreich F, Sindern E, Lorenz R, Adams HA, et al. Educational campaign on stroke in an urban population in Northern Germany: influence on public stroke awareness and knowledge. International Journal of Stroke. 2013;8(5):286-92.

67. Dopson S, Locock L, Gabbay J, Ferlie E, Fitzgerald L. Evidence-based medicine and the implementation gap. Health:. 2003;7(3):311-30.

68. Morgenstern LB, Bartholomew LK, Grotta JC, Staub L, King M, Chan W. Sustained benefit of a community and professional intervention to increase acute stroke therapy. Archives of Internal Medicine. 2003;163(18):2198-202.

69. Greiner AC, Knebel E. Health professions education: A bridge to quality: National Academies Press; 2003.

70. Akinyemi RO, Owolabi MO, Adebayo PB, Akinyemi JO, Otubogun FM, Uvere E, et al. Task-shifting training improves stroke knowledge among Nigerian non-neurologist health workers. Journal of the Neurological Sciences. 2015;359(1-2):112-6.

For peer review only - http://bmjopen.bmj.com/site/about/guidelines.xhtml

Table 1: Distribution of interview participants and study hospitals

Participants	Tertiary/Teaching Hospitals	Regional Hospitals	Total
Nurses	11	9	20
Medical Doctors/Physicians	6	6	12
Clinical Psychologist	1	1	2
Physiotherapist	2	3	5
Dietitian	0	1	1
**Total**	**20**	**20**	**40**

For peer review only

Table 2: Themes and definitions

Coding Categories	Definition of Barriers
Patient level	Includes factors, such as late arrival or low awareness of stroke symptoms, denial of stroke, financial capacity, socio-cultural practices or beliefs inhibiting access or adherence to optimal acute stroke care.
Hospital or health system level	Relates to a lack of inadequate medical facilities or equipment, staff numbers, protocols, management support, supporting policies, organisational context or norms which support implementation of standard care and availability of staff professional development opportunities to support the provision of standard care
Stroke care professionals	Describes acute stroke care providers' level of team support, communication or collaborations which affect the provision of care. Also includes competence, skill, knowledge, awareness, familiarity or agreement to specific treatments, their values, motivations or attitudes towards particular treatments or intervention
National /state health policy context	Relates to the level of political will for acute stroke care in the form of national stroke policies, limited allocation of resources for acute stroke care, reimbursement of funds to hospitals, national health policies to support stroke patients access to optimal care and the lack of any regulatory frameworks or policies to support stroke care.

For peer review only

For peer review only - http://bmjopen.bmj.com/site/about/guidelines.xhtml

Supporting File 1: Consolidated criteria for reporting qualitative studies (COREQ): 32-item checklist

No. Item	Guide questions/description	Reported on Page #
**Domain 1**: **Research team and reflexivity**		
Personal Characteristics		
1. Interviewer/facilitator	LB and AdGA facilitated the recruitment process. LB conducted the interviews	Page 7<br>Line 175-176, 186,
2. Credentials	LB (PhD Candidate), AdGA (PhD), GM (PhD), AS (PhD), CKYC (PhD), SS (PhD)	Page 6-7<br>Lines 155-164
3. Occupation	LB (Public Health PhD Candidate), AdGA (Professor of Social Psychology), AS (Health Services Researcher and Public Health Lecturer), GM (Epidemiologist and Public Health Lecturer), CKYC (Senior Lecturer in Health Psychology, Knowledge dissemination and implementation researcher) SS (Associate Professor of Public Health)	Page 6-7<br>Lines 155-165
4. Gender	Four researchers were males and two were females	Page 7<br>Lines 165-166
5. Experience and training	LB, AdGA, and AS have extensive experience in conducting qualitative research studies. GM, CKYC, SS have relevant training and knowledge in qualitative research studies.	Page 6-7<br>Lines 155-164
Relationship with participants		
6. Relationship established	No	Page 6-7<br>Lines 155-166
7. Participant knowledge of the interviewer	The interviewer (LB) is familiar with some of the study contexts through previous works so it is possible some participants have met or are aware of their works.	Page 6<br>Lines 155-158
8. Interviewer characteristics	The interviewer (LB) is a health services researcher with interest in health services and policy research, research on implementation science and quality improvement interventions for stroke care health professionals. He is skilled in both qualitative and quantitative research works	Page 6<br>Lines 155-160
Domain 2: study design		
Theoretical framework		
9. Methodological orientation and theory	Thematic analysis and Grounded theory	Page 8<br>Lines 200
Participant selection		
10. Sampling	Purposive sampling	Page 7<br>Line 175
11. Method of approach	Participants were first made aware of the study by their ward in charges, head of department, health services administrators, in-	Page 7

For peer review only

	service training and research coordinators, human resource managers and medical directors.	Lines 177- 182
12. Sample size	40	Page 7<br>Please see Table 1
13. Non-participation	Due to time and workload factors, three participants declined to be interviewed	Page 7<br>Lines 182-183
Setting		
14. Setting of data collection	Medical wards, conference rooms and staff office rooms and patient consulting rooms	Page 7-8<br>Lines 187-189
15. Presence of non-participants	No	
16. Description of sample	This has been outlined in Table 1 and the section on participants	Page 7<br>Lines 168- 173
Data collection		
17. Interview guide	An interview guide and prompts facilitated the interview process. The guide was drafted, pilot tested and consequently revised. See supplementary file 1	Pages 7-8<br>Lines 189-192
18. Repeat interviews	No	
19. Audio/visual recording	Yes interviews were audio recorded	Pages 8<br>Lines 192-193
20. Field notes	Yes	Page 8<br>Line 193
21. Duration	30-60minutes	Page 8<br>Lines195-196
22. Data saturation	Yes	Page 7<br>Lines 183-184
23. Transcripts returned	Yes but only 13 participants replied with their comments	Page 8<br>Line 196-198
Domain 3: analysis and findings		
Data analysis		
24. Number of data coders	1	Page 8<br>Lines 203-204
25. Description of the coding tree	No	
26. Derivation of themes	Themes were both identified in advance and emerged from the data	Page 8<br>Lines 201-204

For peer review only

27. Software	NVivo version 10.0	Page 8<br><br>Line 209
28. Participant checking	No	
Reporting		
29. Quotations presented	Yes	Pages 9-17
30. Data and findings consistent	Yes	Pages 9-17
31. Clarity of major themes	Yes	Page 9 and 30
32. Clarity of minor themes	Yes, minor subthemes were presented	Pages 9-17, 30

For peer review only

Supplementary File 2: Characteristics of study hospitals

Hospital	2014 stroke admissions	Hospital bed capacity
TH1	1500	653
TH2	1000	650
TH3	118	500
RH4	409	194
RH5	520	235
RH6	49	200

TH =Tertiary (Teaching) Hospital,

RH= Regional Hospital

For peer review only

Supplementary File 3: Interview Guide

a. Let's start with a brief explanation of what you do in this hospital in terms of stroke care? Kindly explain to me what you are expected to do when a stroke patient has been transferred or report to the hospital with a stroke-like symptoms?

b. What different acute stroke care services or treatments are provided for the care of acute stroke patients in this hospital? (Probe for the awareness and use of stroke unit care, aspirin therapy or thrombolytic therapy, etc.)

c. Do you perceive the current acute stroke care services and therapies for stroke patients as helpful in providing care or there are some challenges in using them?

d. How is acute stroke care provided in this hospital? (Probe to understand if the provision of care is guided by clinical guidelines or protocols and if so, what types of guidelines or protocols are used?)

e. Do you find these guidelines helpful in providing care or face some challenges in trying to use them? Where they exist, probe on the following: (their clarity and relevance to stroke clinical care, stroke care professionals familiarity with and confidence in clinical guidelines usage, their attitudes towards clinical guidelines and the perceived barriers of these guidelines in clinical decision-making

f. Now let's discuss the current practical challenges which hinder the delivery of optimal care to stroke patients? Could you elaborate on some of the barriers you face on daily bases? Probe on the following: Guideline factors, health staff level barriers, patient factors, incentives and resources, policy decisions/contexts, national level factors, etc.

g. How do you cope or manage to provide stroke care in the midst of such barriers?

h. What recommendations will you like to make to the hospital authorities on how to improve acute stroke care in the hospital?

i. Is there anything you will like to share, either audio-recorded or off audio recorded in relation to the issues we have just discussed?

j. I will be transcribing the recording and if you don't mind I will be happy to share the interview transcript with you to cross check to be sure what is transcribed reflects your views?

k. Thank you for your time

For peer review only
